# Coexpression of Nuclear Receptors and Histone Methylation Modifying Genes in the Testis: Implications for Endocrine Disruptor Modes of Action

**DOI:** 10.1371/journal.pone.0034158

**Published:** 2012-04-04

**Authors:** Alison M. Anderson, Kim W. Carter, Denise Anderson, Michael J. Wise

**Affiliations:** 1 Computer Science and Software Engineering, University of Western Australia, Perth, Australia; 2 Telethon Institute for Child Health Research, Centre for Child Health Research, University of Western Australia, Perth, Australia; 3 Biomedical, Biomolecular and Chemical Sciences, University of Western Australia, Perth, Australia; Institut Jacques Monod, France

## Abstract

**Background:**

Endocrine disruptor chemicals elicit adverse health effects by perturbing nuclear receptor signalling systems. It has been speculated that these compounds may also perturb epigenetic mechanisms and thus contribute to the early origin of adult onset disease. We hypothesised that histone methylation may be a component of the epigenome that is susceptible to perturbation. We used coexpression analysis of publicly available data to investigate the combinatorial actions of nuclear receptors and genes involved in histone methylation in normal testis and when faced with endocrine disruptor compounds.

**Methodology/Principal Findings:**

The expression patterns of a set of genes were profiled across testis tissue in human, rat and mouse, plus control and exposed samples from four toxicity experiments in the rat. Our results indicate that histone methylation events are a more general component of nuclear receptor mediated transcriptional regulation in the testis than previously appreciated. Coexpression patterns support the role of a gatekeeper mechanism involving the histone methylation modifiers *Kdm1*, *Prdm2*, and *Ehmt1* and indicate that this mechanism is a common determinant of transcriptional integrity for genes critical to diverse physiological endpoints relevant to endocrine disruption. Coexpression patterns following exposure to vinclozolin and dibutyl phthalate suggest that coactivity of the demethylase *Kdm1* in particular warrants further investigation in relation to endocrine disruptor mode of action.

**Conclusions/Significance:**

This study provides proof of concept that a bioinformatics approach that profiles genes related to a specific hypothesis across multiple biological settings can provide powerful insight into coregulatory activity that would be difficult to discern at an individual experiment level or by traditional differential expression analysis methods.

## Introduction

Endocrine disruptors are a highly heterogeneous group of compounds that range from synthetic chemicals and pesticides to natural compounds found in foods [1]. Results from animal studies have linked exposure to these compounds with adverse health outcomes ranging from reproductive [2] and metabolic disorders [3] to alterations in neuronal development [4]. Perturbation of hormonal systems such as receptor binding, hormone production, release, transport or elimination is well documented [1], [5]. Less well understood and of growing concern is the potential for these compounds to perturb epigenetic mechanisms during embryogenesis and thus increase the risk of dysfunction and disease susceptibility later in life.

The OECD (Organisation for Economic Co-operation and Development) recently published a review entitled *Endocrine Disruptors and the Epigenome* (available at www.oecd.org/dataoecd/42/53/48435503.pdf). The authors state that evidence to date is highly-suggestive of a role for epigenetic dysregulation in the mediation of endocrine disruptor exposure effects. Consequently the incorporation of epigenetic knowledge into chemical safety assessment regulatory activity is now a major objective for the OECD. This objective continues to be challenged by gaps in knowledge surrounding the intersection of endocrinology and epigenetic biology, see Zhang and Ho [6] and LeBaron [7] for pertinent reviews.

Nuclear receptors are an integral component of transcription regulation. The mechanisms that confer context specificity and fine-tuning of nuclear-receptor mediated changes in gene expression continue to be elucidated and are found to be increasingly complex. In addition to the classical ligand mechanism, ligand-independent action of receptors is reported [8] together with the involvement of complexes of coactivators and corepressors that encompass groups of molecules with a diverse range of functional capacity. The work described here focuses on the subset of coregulators that are associated with one specific aspect of the epigenetic apparatus: the modification of histone methylation. The term *coactivity* is used herein to describe the sequential and combinatorial regulatory action of nuclear receptors and histone methylation modifiers within the context of transcriptional regulation.

Histone methylation plays a role in the establishment and maintenance of long-term epigenetic marks such as X chromosome inactivation and imprinting [9] and also in chromatin modification associated with temporal transcriptional changes [10], [11]. A role for histone methylation events has been described in processes susceptible to endocrine disruption such as steroidogenesis [12], spermatogenesis [13] and embryonic development [11]. Functional studies that knock out or reduce the levels of enzymes that modify histone methylation status describe phenotypic outcomes similar to those observed following exposure to endocrine disruptor compounds [14]. Increasingly, reports in the literature link histone methylation and nuclear receptor activity [13], [15]–[17]. Of particular interest are a gatekeeper mechanism reported by Garcia-Bassets et al [18], whereby histone methylation events prevent constitutive expression in the absence of ligand, and nuclear receptor interaction with components of histone methylation modifying complexes - collectively referred to herein as ASCOM (ASC-2 [activating signal cointegrator-2] complexes [19]–[22]. The nuclear receptor interacting protein NCOA6 (also named activating signal cointegrator-2 (ASC-2)), has been shown in mammalian cells to complex with ASCOM and to act as a linker protein between nuclear receptors and ASCOM components [21]. Direct interaction between ASCOM components and estrogen [22]–[24], progesterone [25] and peroxisome proliferator-activated receptors [24], [26] has also been reported. Together, this evidence led us to hypothesise that histone methylation events may be susceptible to perturbation by endocrine disruptor compounds via their coregulatory activity with nuclear receptors. We envisaged that publicly available gene transcription data could be analysed to gain insight into coregulatory activity and susceptibility of these mechanisms to compound exposure.

A limitation of transcriptomics is that only a snap shot is captured of either perturbed or normal function. The common toxicogenomics approach whereby pathways perturbed following exposure are inferred by statistical enrichment of top differentially expressed genes is not always optimal. Subtle changes in the levels of mRNA for some genes may have strong biological consequence, but may be missed or overshadowed by larger changes occurring in the many genes affected at a whole genome level. Also, given that functional investigations of the epigenome are recently published, this aspect of biology is likely to be currently under-represented in the gene ontology annotations commonly used as a basis for enrichment analysis. Furthermore, given the temporality of histone methylation events and the interdependency of genes within coregulatory complexes, analysis of changes in simple binary associations following exposure is likely to be misleading.

With these limitations in mind we opted to predefine an analysis geneset enriched for our study objectives and profile both expression and coexpression within this geneset in a range of normal and exposure settings.

The intersection between nuclear receptor activity and the histone methylation aspect of the epigenetic apparatus has several implications for endocrine disruptor mode of action. Firstly there is the possibility that compound-induced alteration of histone methylation activity is a common contributing factor to the dysregulation of genes with diverse phenotypic outcomes and thus an explanatory factor in multiple phenotypic outcomes from exposure to a single compound. The estrogenic endocrine disruptor diethylstilbestrol is a case in point where exposure in animal studies has been associated with a range of cancers, malformation of the genital track, obesity [27] and at a molecular level changes in DNA methylation [28]. Secondly there are implications for direct or indirect long-term effects arising from epimutations or aberrant epigenetic function. Consider, for example, the dysregulation of genes on the steroidogenic pathway following *in utero* exposure to some phthalates. This temporal perturbation indirectly contributes in part to malformations in offspring through disruption of hormonal activity at critical developmental time points [5]. From an epigenetic perspective, it is feasible that compound exposure elicits similar temporal effects in histone methylation balance that indirectly result in long-term effects. Alternatively exposure may give rise to permanent aberrant methylation marks that alter the response of affected genes to future environmental stimuli and thus increase susceptibility to dysfunction or disease later in life. The rationale behind the current investigation is that both short-term, indirect long-term and direct long-term effects might share a common etiology that involves, in part, nuclear-receptor mediated changes in histone methylation status.

We used publicly available expression data to appraise whether and to what extent nuclear receptors show coexpression with genes involved in histone methylation modification in testis tissue across a range of biological settings including species, development stage and following exposure. In regards to exposure it should be clarified that the objective of the study was *not* to identify correlation between compound-specific exposure and coexpression patterns as this would require alternative study designs. Rather, our objective was to identify coexpression patterns that support the concept of nuclear receptor coactivity with histone methylation modifying genes. The rationale being that these mechanisms represent a pathway between nuclear receptor action and the modification of developmentally critical epigenetic marks and such mechanisms could provide insight into aspects of endocrine disruption that remain enigmatic.

Similar coexpression patterns between specific nuclear receptors and histone methylation modifiers in testis tissue from different species and across experiments were observed, strengthening the evidence for coactivity and or interdependence between these two distinct functional gene groups. Importantly, our results are a proof of concept that profiling a set of genes related to a specific hypothesis across multiple biological settings can identify patterns of coregulatory dynamics that are difficult to discern at an individual experiment level. These patterns provide a framework against which endocrine disruption can be best analysed and better understood.

## Results

### In silico validation of enriched geneset

As detailed in Methods and Table S1, the enriched geneset consisted of groups of genes belonging to the nuclear receptor family, histone methylation modifiers and genes key to endocrine disruption phenotypes. As an additional validation technique, we analysed our selected geneset to verify that the members show enrichment for our hypothesis. We performed canonical pathway enrichment as implemented in the Ingenuity Pathway Analysis (IPA) software (Ingenuity© Systems, www.ingenuity.com). Results from this analysis confirmed that 74% (102/137) of the genes in the analysis set were functionally associated with transcriptional regulation. Physiological systems identified by canonical pathway enrichment included endocrine system development and function (31 genes), embryonic development (62 genes) and regulation of development (81 genes).

### Coexpression analysis across biological settings

Coexpression analysis between nuclear receptors and histone methylation modifiers in testis tissue across multiple experiments provided insight into the generality of their coactivity: all of the nuclear receptors (n = 41) were observed to show strong coexpression (Pearson *r*≥0.75 or ≤−0.75, p-value<0.05) with one or more histone methylation modifiers in the normal testis tissue of one or more species. Coexpression relationships of similar strength involving 12 nuclear receptors, 16 methyltransferases and three demethylases, were observed in all three species from Experiment 1, see Table S2 A. 21% (90/421) of the coexpression relationships observed in rat testis of this experiment were also observed with similar strength and direction in one or more of the spermatocyte or round spermatid samples from Experiment 10. Across the seven studies in rat 62 coexpression relationships involving 23 nuclear receptors and 35 histone methylation modifiers were observed three or more times in normal tissue samples Most represented in this group were the nuclear receptors *Nr2c1* (26 occurrences), *Nr3c1* (16 occurrences) the demethylases *Kdm3a* (13 occurrences) and *Kdm1* (12 occurrences) and the methyltransferases *Cxxc1* (13 occurrences) and *Setdb1* (12 occurrences).

Experiment 7 samples represented embryonic tissue collected from the efferent ducts, epididymis and vas deferens. Correlated expression of similar strength and direction between the nuclear receptor *Rarb* and the methyltransferase *Suv39h1* and the nuclear receptor *Nr1h2* and the methyltransferase *Suv420h2* was observed in all three of these tissues. A further 49 coexpression relationships were observed in two of the three tissues, see Table S3 A. Results from Experiment 7, 8 and 9, which represent tissues from whole testis, revealed 115 coexpression relationships to be conserved across all embryonic mouse tissue, see Table S3 B. Most represented in this group were the nuclear receptors *Ppard* (25 occurrences) and *Nr4a2* (18 occurrences), the demethylase *Kdm2b* (9 occurrences) and the methyltransferase *Ehmt2* (19 occurrences). Comparison between embryonic and adult tissue in mouse revealed 47 conserved coexpression relationships and only 18 coexpression relationships were conserved between mouse and rat embryonic tissue.

The observation of similar coexpression patterns between specific nuclear receptors and histone methylation modifiers in testis tissue from different species and across experiments strengthens the evidence for coactivity and or interdependence between these two distinct functional gene groups.

### ASCOM (Activating Signal Cointegrator-2 complex)

**Figure 1:**
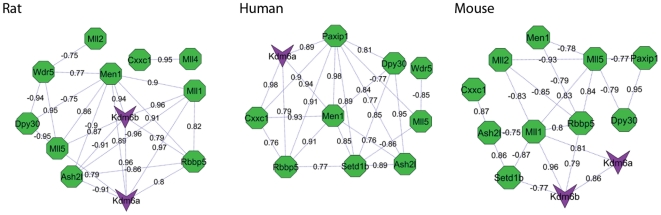
Coexpression patterns in human, mouse and rat testis. Coexpression between ASCOM genes in rat human and mouse are represented as networks. Green octagons = methyltransferases and purple vee = histone demethylases. Edge labels represent Pearson correlation significant at p<0.05.

ASCOM represents a pool of complexes – each of which contain an evolutionarily conserved core – comprising the WD repeat domain 5 (WDR5) protein, the ASH2-like protein (ASH2L) and the retinoblastoma binding protein 5 (RBBP5) and one or more additional methyltransferase with specificity for the H3K4 substrate [21]. The methyltransferases MLL1 to MLL5, MEN1, CXXC1, PAXIP1, DPY30 and the demethylases KDM6A and KDM6B have also been reported as components of ASCOM and ASCOM like histone methylation modifier complexes. Coactivity of these proteins was reflected in our IPA network analysis, see Figure S1. The functional relevance of these complexes in testis tissue is yet to be explored. Here, genes encoding these proteins were found to have strongly correlated expression patterns in the testis tissue of all three species, see Figure 1. The protein transcribed from *Ncoa6* is considered to act as an integrator between nuclear receptors and ASCOM complexes. 85% (35/41) of the receptors in the analysis geneset showed correlated expression with *Ncoa6* across the group of all normal samples. In grouped exposed samples 73% (30/41) showed correlated expression with *Ncoa6* and 18 of the nuclear receptors were common to both groups.

*Ncoa6* showed strong coexpression with all ASCOM components with the exception of *Mll3* and *Mll4* in both normal and exposed tissue, eight of the coexpression relationships were observed in three or more sample sets. These results indicate that recruitment of the ASCOM complex is a more general component of nuclear receptor mediated transcriptional regulation in the testis than previously appreciated.

### Gatekeeper mechanism

**Figure 2:**
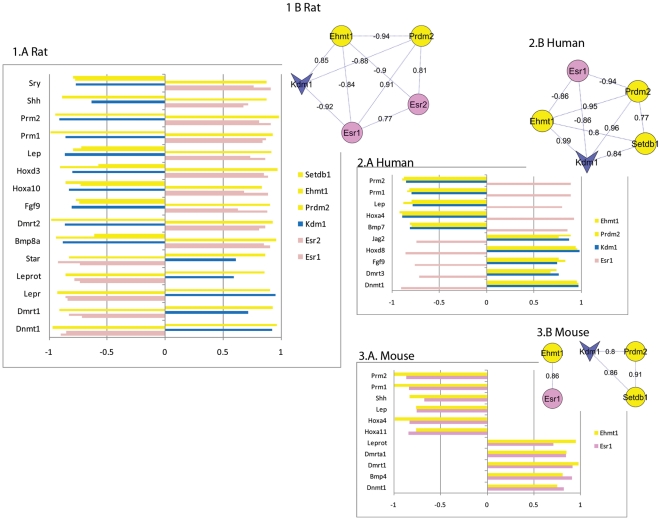
Gatekeeper gene coexpression patterns across species. Coexpression between genes implicated in the gatekeeper mechanism described by Garcia_Bassets et al are represented as networks. Pink ellipses = nuclear receptors, yellow = histone methyltransferases and blue vee = histone demethylase. Edge labels = Pearson correlation significant at p<0.05. These coexpression relationships are reflected in the coexpression patterns between gatekeeper genes and genes relevant to endocrine disruptor phenotypes and the methyltransferase *Dnmt1*, shown in associated bar graphs for rat, human and mouse Bar lengths represent Pearson correlation, positive values = positive correlation and negative values = inverse correlation, significant at p<0.05. Genes relevant to endocrine disruptor phenotypes: sexual differentiation (*Bmp7*, *Bmp8a*, *Dmrt1*, *Dmrt2*, *Dmrt3*, *Dmrta1*, *Fgf9*, *Jag2*, *Shh*, *Sry*); embryonic development (*Bmp4*, *Hoxa4*, *Hoxa10*, *Hoxa11*, *Hoxd3*, *Hoxd8);* obesity (*Lep*, *Lepr*, *Leprot*); spermatogenesis (*Prm1*, *Prm2*) and steroidogenesis (*Star*).

Garcia-Bassets et al [18] identified a role for histone modification as a gatekeeper mechanism that acts to either facilitate promoter access to liganded-nuclear receptors or to prevent access and thus constitutive recruitment of receptors to promoters in the absence of ligand. Specifically, they showed that, in the presence of liganded-receptors, recruitment of the demethylase KDM1 to the promoter of some estrogen receptor target genes was required to remove repressive marks conferred by the methyltransferases EHMT1, PRDM2 and ESET (also known as SETDB1). To identify a potential role for this mechanism in endocrine function in the testis, we profiled expression patterns between estrogen receptors, the gatekeeper methylation modifiers identified by Garcia-Bassets et al, and genes in our analysis set that were relevant to endocrine disruptor phenotypes. In the rat data, both estrogen receptors showed coexpression with *Ehmt1* and *Prdm2*, and *Esr1* was negatively coexpressed with *Kdm1*. A consistent pattern between the estrogen receptors, the methylation modifiers and 64% (14/22) of the phenotypic genes was also observed. In human samples, only the estrogen receptor alpha (*Esr1*) showed coexpression with the gatekeeper genes and a similar pattern of coexpression with nine phenotypic genes was observed. An interesting pattern was observed in the mouse data whereby only one gatekeeper gene *Ehmt1* showed coexpression with *Esr1* and this relationship was reflected in the coexpression patterns of ten phenotypic genes. Together these patterns support the concept of a gatekeeper mechanism involving *Esr1* and histone methylation modifiers in testis tissue. Figure 2 provides a visual representation of these coexpression patterns whereby the coexpression between genes involved in the gatekeeper mechanism are shown as a network and the strength of coexpression of these genes with each phenotypic gene is represented in a bar graph.

**Figure 3:**
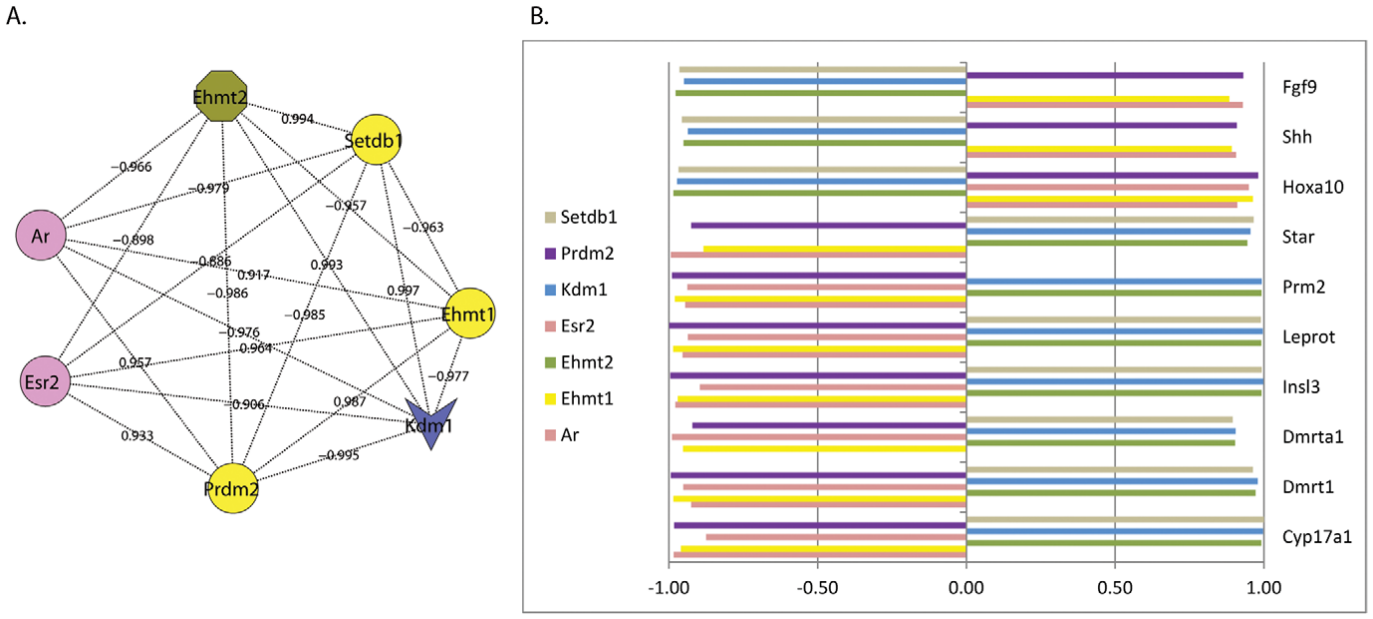
Gatekeeper gene coexpression patterns from Experiment 6. **A:** Coexpression between genes implicated in the gatekeeper mechanism identified by Garcia- Bassets et al are represented as a network. Pink ellipses = nuclear receptors, yellow and olive green = histone methyltransferases and blue vee = histone demethylase. Edge labels = Pearson correlation significant at p<0.05. **3.B:** The coexpression relationships in **3.A** are reflected in the coexpression patterns between gatekeeper genes and genes relevant to endocrine disruptor phenotypes: sexual differentiation (*Dmrt1*, *Dmrta1*, *Fgf9*, *Shh*, *Insl3*); embryonic development (*Hoxa10*), *steroidogenesis (Cyp17a1, Star);obesity (Leprot) and* spermatogenesis (*Prm2*).

The reflection of nuclear receptor histone methylation coexpression patterns in the coexpression patterns of phenotypic genes was evidenced in several of the data sets. A clear example was observed in data from Experiment 6. This dataset contained samples obtained from controls and from rats exposed from gestational day six to postnatal day 92 to low and high dose myclobutanil, low and high dose propiconazole and low and high dose triadimefon. The observed pattern involved the *Ar* and *Esr2* receptors, the methyltransferases *Ehmt2*, *Ehmt1*, *Prdm2* and *Setdb1* and the demethylase *Kdm1*. The pattern is best understood as having two components the first of which represents the coexpression profile of the coregulatory genes, henceforth referred to as the *gatekeeper set* (*Esr2*, *Ehmt2*, *Ehmt1*, *Kdm1*, *Prdm2* and *Setdb1*). The second component represents the coexpression between phenotypic genes and genes in the gatekeeper set. In samples that had been exposed to high dose triadimefon, the gatekeeper set were found to have highly correlated expression patterns, shown as a network in Figure 3. In the same samples, of the 22 phenotypic genes in the analysis geneset, nine did not show coexpression with either *Ar* or *Esr2*, three had coexpression approaching significance with *Esr2* and 10 had strong coexpression with *Esr2*, *Ar* or both. In the latter group the 10 phenotypic genes also showed strong coexpression with the remaining members of the gatekeeper set in a consistent pattern of correlation or anti-correlation, see Figure 3 bar graph. Phenotypic genes with coexpression approaching significance showed a similar pattern with the gatekeeper set genes that was also approaching significance and genes found not to have correlated expression with either of the nuclear receptors showed no correlated expression with any of the gatekeeper genes.

By comparison, in samples exposed to low levels of propiconazole only two of the gatekeeper genes showed high coexpression with each other and none showed coexpression with a nuclear receptor. In these samples none of the 22 phenotypic genes showed coexpression with any components of the gatekeeper geneset.

These patterns observed in Experiment 6 further support the concept of the gatekeeper mechanism whereby histone methylation modifiers work in concert with nuclear receptors to mediate transcriptional change at target genes. The observation of such patterns in both normal and exposed tissue indicate that this coactivity is not specific to either normal function or exposure response but likely represents a more general component of transcriptional regulation. From the perspective of endocrine disruption the main point of interest is that gatekeeper type mechanisms may represent a common regulatory mechanism for genes with diverse functional roles and thus a potential explanatory factor for compound exposure that elicits multiple phenotypic outcomes.

### ASCOM coexpression patterns in controls and exposed samples

In the data from experiments 2–4, which all involved *in utero* exposure, *Ncoa6* was observed to have correlated expression with a higher number of genes in the analysis geneset in exposed samples (n = 62) as compared to controls (n = 21). This suggests that recruitment and activity of ASCOM and other transcriptional regulatory complexes associated with *Ncoa6* increases overall following *in utero* compound exposure. The specific actions of histone methylation modifiers is determined, in part, by the proteins they complex with, so while the number of genes *Ncoa6* showed correlated expression with was similar in both controls (n = 9) and exposed (n = 8) samples in the adult exposure setting of Experiment 5, the function of the coexpressed genes suggest that different transcriptional outcomes would have been elicited. In the control samples, *Ncoa6* showed strong coexpression with ASCOM methyltransferases that confer marks associated with transcriptional activity (*Mll1 r* = 0.89, *Mll4 r* = 0.89, *Mll5 r* = 0.90), while in exposed samples positive coexpression with the corepressors *Hdac2* (*r* = 0.90) and *Ncor2* (*r* = 0.91) was observed.

*Wdr5* is a core structural component of ASCOM which plays a critical role in substrate recognition and bridging of catalytic components to the histone tail [29], [30]. We found increased correlation of this gene in the group of exposed samples (n = 168) (n = 57) as compared to the group of controls (n = 21) (n = 56) from the same experiments. An increase in coexpression between Wrd5 and other methyltransferases indicates overall global changes in histone methylation. Resulting transcriptional outcomes would be substrate and target gene specific.

Interestingly, in control samples of Experiment 3 *Wdr5* showed strong coexpression with the gatekeeper gene *Prdm2* (*r* = 0.98), which confers a silencing mark, and was not observed to have coexpression with any other ASCOM components in this setting. In contrast, when strongly coexpressed with the gatekeeper activator *Kdm1* in exposed (dibutyl phthalate) samples from the same experiment (*r* = 0.99), vinclozolin exposed samples from experiment 2 (*r* = 0.93) and a coexpression relationship approaching significance in dibutyl phthalate exposed samples from experiment 4 (*r* = 0.94, p = 0.06), correlated expression with one or more ASCOM components was observed. In each of these exposure settings simultaneous coexpression with an estrogen receptor was also observed.

A similar pattern was observed in Experiment 6. *Wdr5* showed correlated expression with the highest number of ASCOM components in the samples exposed to high levels of triadimefon (n = 11) in comparison to the five other exposure settings (n = 0 or 1) and controls (n = 4). *Wdr5* also showed correlated coexpression with *Kdm1* and anti-correlated expression with *Esr2* in both the control and high triadimefon exposure settings and in the latter strong anti-correlation with *Prdm2* was also observed.

**Figure 4:**
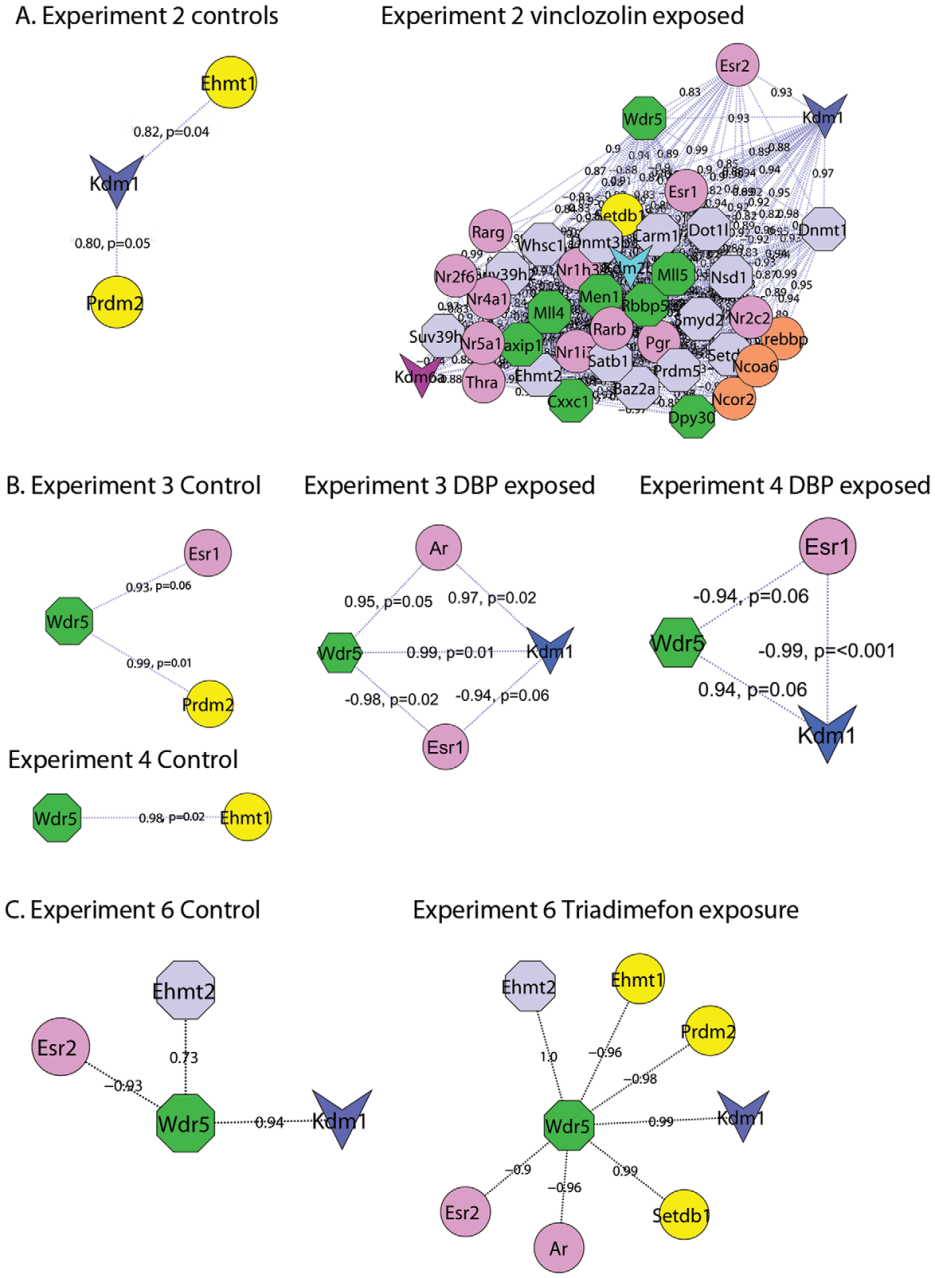
Coexpression between gatekeeper genes and ASCOM component *Wdr5*. Coexpression patterns observed in both controls and exposed samples support a role for a gatekeeper mechanism. Given the dynamic nature of histone methylation, perturbation cannot be inferred from differences between control and exposed groups. More in-depth studies are warranted to investigate exposure effects on these mechanisms. **A:** In control samples from Experiment 2 *Kdm1* was observed to have coexpression higher than the study cut-off limits with only three other histone modifiers including the gatekeeper genes *Ehmt1 and Prdm2*(approaching significance), and no nuclear receptors. In contrast, in samples from the same experiment that are derived from decedents of an F0 vinclozolin exposed dam, *Kdm1* showed strong coexpression with *Esr2*, multiple other nuclear receptors and histone modifiers including the core ASCOM component *Wdr5* and the methyltransferase *Dnmt1*. **B:** In Experiments 3 and 4 *Wdr5* is anti-correlated with *Esr1* and has correlated expression with *Kdm1*. In control samples of Experiment 3 *Wdr5* shows coexpression approaching significance with *Esr1* (p = 0.06) and the gatekeeper gene *Prdm2*. In Experiment 4 only one gatekeeper gene *Ehmt1* shows coexpression with *Wdr5*. **C:** In Experiment 6 *Wdr5 showed similar coexpression with Esr2, Ehmt2 and Kdm1* in both control and exposed samples and with additional gatekeeper genes in the exposed group. Pink ellipses = nuclear receptors, green = histone methyltransferase and blue = histone demethylase. Edges represent Pearson correlation; some of these relationships were approaching significance as indicated by the associated p-values.

In addition to its role in the gatekeeper mechanism *Kdm1* is implicated in coactivation of the androgen receptor via modification of H3K9me2/me1 [16]. In Experiment 3 the androgen receptor showed positive coexpression with *Kdm1* (*r* = 0.97) and coexpression approaching significance with *Wdr5* (*r* = 0.95, p = 0.05), see Figure 4.

Observed coexpression between the gatekeeper gene *Kdm1* and core ASCOM component *Wdr5* in multiple settings suggest that these mechanisms act in concert to confer nuclear receptor mediated transcriptional regulation. Coexpression between these genes and androgen and estrogen receptors in multiple settings indicate that their coactivity is a potential point of convergence between estrogen and androgen signalling pathways. Changes in their coexpression patterns following exposure suggest a role in toxicity response, however given the temporality of histone methylation events it cannot be discerned from these patterns alone as to whether these changes represent normal or aberrant function.

### A role for the gatekeeper and ASCOM complexes in Star gene dysregulation

Perturbation of the steroidogenic pathway is a known mode of action for several endocrine disruptor compounds [5]. The steroidogenic acute regulatory protein StAR is the rate limiting protein in steroidogenesis and epigenetic regulation of this gene has been previously described by Hiroi et al [12]. This work provides a background against which changes in coexpression observed in the current study can be best interpreted. Hiroi et al describe multiple substrate and gene loci specific alterations to histone marks during and following hormone induced transcriptional changes. In particular, they noted a reduction in the repressive mark H3K9me2, initially in the promoter region and then in the coding region of the *Star* gene, that was associated with hormone-induced increased transcription in MA-10 and mouse granulose cells. Here, coexpression patterns between the *Star* gene and histone methylation modifiers varied across settings and between control and exposed samples. Common to more than one exposure setting, however, were changes in coexpression with the gatekeeper methyltransferase *Prdm2* which has specificity for H3K9me2. This gene was observed to have negative correlation with the *Star* gene in both *in utero* exposures (vinclozolin *r* = −0.87, dibutyl phthalate *r* = −0.95) and a negative correlation approaching significance was also observed in testis samples from rats exposed to dibutyl phthalate (*r* = −0.84, p = 0.075) at age10 weeks. The ASCOM component *Men1* was observed to have correlated expression with the *Star* gene in control samples collected at gestational day 20 (*r* = −0.99) from Experiment 3, control samples collected at embryonic day 16 (*r* = 0.99) from Experiment 2 and in the normal samples of experiment 1 (*r* = 0.87). *Men1* confers the H3K4me3 mark associated with gene activation and has recently been linked to estrogen dependent coactivation [31]. Loss of correlated expression between this gene and the *Star* gene in exposed samples may be a contributing factor to dysregulation of *Star* expression. The coexpression patterns observed between *Prdm2* and *Men1* with the *Star* gene, observed in multiple studies, further suggest that the gatekeeper and ASCOM complexes work in concert as estrogen receptor mediated transcriptional regulators.

### Dnmt1 and the gatekeeper mechanism

The DNA (cytosine-5-)-methyltransferase *Dnmt1* has been observed to influence the ratio of H3K4me2/H3k9me2 [32] in addition to its role as a cytosine methyltransferase. Here, *Dnmt1* showed the same coexpression pattern with the gatekeeper genes as was observed with phenotypic genes in normal tissue samples from Experiment 1, see Figure 2. These patterns indicate a role for the gatekeeper mechanism in transcriptional regulation of *Dnmt1* in the testis.

### Kdm1 and Dnmt1coexpression patterns

*Kdm1* has been previously reported to demethylate and stabilise *Dnmt1*, and is purported to act as a link between the histone and DNA methylation systems [33]. In the vinclozolin experiment *Kdm1* and *Dnmt1* were strongly correlated and showed similar changes in coexpression patterns following exposure. Both genes had increased coexpression with transcriptional modifiers in exposed samples (*Kdm1* n = 41, *Dnmt1* n = 42) as compared to controls (*Kdm1* n = 2, *Dnmt1* n = 11). Common to the coexpression hubs of *Kdm1* and *Dnmt1* in exposed samples were 38 relationships involving core and catalytic components of the ASCOM complex and nine nuclear receptors including both estrogen receptors, *Nr5a1* (also known as steroidogenic factor SF-1) and *Nr2c2*, which is required for normal spermatogenesis and early embryonic development, see Figure 4

The coexpression patterns observed in the vinclozolin data indicate that multiple nuclear receptors and histone methylation modifiers act in concert and their coactivity has the potential to influence both histone and DNA epigenetic marks.

### Differential expression

Differential expression of all genes in the analysis geneset showed very few genes to be dysregulated in each experiment at the study cut-off limits. Of all the exposure settings of Experiment 6, only the high dose triadimefon showed dysregulation and only modest fold changes for two genes (*Cyp17a1* and *Insl3*) which were not significant after adjusting for multiple testing. No histone methylation modifying genes were observed to be dysregulated in dibutyl phthalate Experiments 3–5. In Experiment 3, comparison between controls and vinclozolin exposed samples for each generation (controls n = 2, exposed n = 2) showed the demethylase *Kdm6b* to be down-regulated at each generation and the gatekeeper gene *Ehmt1* showed statistically significant down-regulation in each generation but just below the study cut-off value of a 1.5 fold change. When the samples were grouped and analysed as controls (n = 6) versus vinclozolin exposed (n = 6), differential expression of methylation modifiers and nuclear receptors was more evident, see Tables 1 and 2, however differential expression patterns did not reflect the changes observed in the coexpression analysis. These results indicate that histone methylation events and changes in the complex dynamics of coregulatory genes involve subtle alterations in mRNA levels that would be difficult to detect in studies with low sample numbers.

**Table 1:** Genes down-regulated in F1–F3 generation following vinclozolin exposure, adjusted p-value<0.05.

Gene Symbol	Description	Fold change
**F1 control v F1 exposed**		
Kdm6b	lysine (K)-specific demethylase 6B	1.79
Rora	RAR-related orphan receptor A	1.61
Nr1h2	nuclear receptor subfamily 1, group H, member 2	1.6
Dnmt3b	DNA (cytsine-5-)-methyltransferase 3 beta	1.53
Baz2a	bromodomain adjacent to zinc finger domain 2A	1.5
Ehmt1	euchromatic histone-lysine N-methyltransferase 1	1.4
**F2 control v F2 exposed**		
Kdm6b	lysine (K)-specific demethylase 6B	1.99
Nr2f2	nuclear receptor subfamily 2, group F, member 2	1.65
Ehmt1	euchromatic histone-lysine N-methyltransferase 1	1.4
**F3 control v F3 exposed**		
Kdm6b	lysine (K)-specific demethylase 6B	2.03
Ehmt1	euchromatic histone-lysine N-methyltransferase 1	1.42

**Table 2:** Genes down-regulated in grouped controls v grouped exposed samples following vinclozolin exposure, adjusted p-value<0.05.

Gene symbol	Description	Fold change
Kdm6b	lysine (K)-specific demethylase 6B	7.06
Nr1d2	nuclear receptor subfamily 1, group D, member 2	3.81
Kdm5b	lysine (K)-specific demethylase 5B	3.76
Rora	RAR-related orphan receptor A	3.14
Nr2f2	nuclear receptor subfamily 2, group F, member 2	2.93
Baz2a	bromodomain adjacent to zinc finger domain 2A	2.66
Ehmt1	nuclear receptor subfamily 1, group H, member 2	2.58
Nr1h2	nuclear receptor subfamily 1, group H, member 2	2.57
Rara	retinoic acid receptor, alpha	2.43
Dnmt1	DNA (cytosine-5-)-methyltransferase 1	2.43
Dnmt3b	DNA (cytosine-5-)-methyltransferase 3 beta	2.27
Nr5a2	nuclear receptor subfamily 5, group A, member 2	2.25
Men1	multiple endocrine neoplasia 1 (menin)	2.18
Ehmt2	euchromatic histone-lysine N-methyltransferase 2	2.13

## Discussion

Recent reviews in the literature describe a critical role for histone methylation marks as determinants of transcriptional potential at the centre of cellular differentiation, long-term epigenetic programming, cellular homeostasis and pathogenesis [9], [11], [12], [19], [34]. Functional studies have identified coactivity between histone methylation modifiers and nuclear receptors but a role for these coregulatory dynamics in testis is yet to be fully elucidated. The activity of enzymes that confer histone methylation events is dynamic, highly context specific and varies according to the coregulatory proteins, including nuclear receptors, that they interact or complex with. Given the temporality and variability in natural function identifying perturbation following exposure is particularly challenging. Here, we trialled a targeted approach whereby the coexpression patterns of a subset of pertinent genes was profiled across testis tissue from a range of biological settings. The coexpression patterns in normal testis tissue provided insight into the generality of coactivity between nuclear receptors and histone methylation modifiers. These data support a role for two specific transcriptional regulatory systems: the gatekeeper mechanism described by Garcia-Bassets et al and recruitment of ASCOM histone modifier complexes.

### Histone methylation modification as both unifying determinant and delineator of endocrine disruption

Endocrine disruptor compounds are reported to elicit a wide range of phenotypic outcomes that are associated with perturbation of nuclear receptor signalling pathways but cannot be adequately explained by this mode of action alone. The coactivity of genes involved in the previously described gatekeeper mechanism, as evidenced in coexpression patterns in the current study, indicate that the gatekeeper concept is common to key genes representing disparate endocrine disruptor phenotypes. Common coexpression patterns with gatekeeper genes in all three species were observed for the protamines *Prm1* and *Prm2* that are essential for chromatin compaction during spermatogenesis, the *Lep* gene which is associated with obesity, the steroidogenic *Star* gene and genes relevant to sexual differentiation and embryogenesis. The research group that identified and described the gatekeeper mechanism also noted that different estrogen receptor target genes are regulated by alternative combinations of histone demethylases and methyltransferases and concluded, as have others, that modification of histone methylation ratios at distinct substrates and gene loci is likely to be a general component of transcriptional regulation. The coexpression patterns observed in the current study further support this concept and indicate that specific combinations of receptors and histone modifiers are promising biomarkers that can discriminate between normal function, exposure response and specific modes of perturbation. Furthermore, identification of common combinations of coactivators and corepressor complexes across biological settings could provide a framework for the principled extrapolation of results across tissue and species, which is an unavoidable and major challenge for health risk assessors.

### Gaining insight into the enigmatic aspects of endocrine disruption

Temporally and spatially inappropriate histone methylation events following exposure could alter long-term transcriptional trajectories and developmental programs via two main modes of action: removal or addition of long-term marks such as H3K27me3 and DNA methylation; or temporary transcriptional perturbation of key genes at critical developmental time points by competing with or altering histone methylation events triggered by normal signalling pathways. Subtle perturbation of histone methylation mark ratios in particular could explain some of the enigmatic features of endocrine disruption such as transgenerational effects and cumulative effects of compounds with disparate modes of action.

### Transgenerational effects

It has recently been shown that mature human spermatozoa retain epigenetic marks such as H3K27 tri-methylation and H3K4 di-methylation at regulatory regions of genes that influence gene expression during male and female gametogenesis and in preimplantation embryos [35]. This research showed that H3K4 di-methylation was associated with low DNA methylation state and, in general, histone marks and DNA methylation – which is required for paternal imprinting in the next generation – were observed to be mutually exclusive [35]. The ASCOM complexes show specificity for the H3K4 and H3K27 substrates. Coactivity between nuclear receptors and ASCOM components, as evidenced by coexpression patterns in this study, suggest a link between hormonal signalling systems with the mechanisms that establish histone methylation marks that are critical to the integrity of epigenetic information passed from one generation to the next. These results indicate that further investigation of nuclear-receptor coactivity with the ASCOM complexes might provide useful insight into transgenerational toxicity modes of action and these mechanisms should be a focus of endocrine disruptor research.

### Cumulative exposure investigations

Compounds that act by disparate mechanisms of toxicity have demonstrated cumulative dose-additive effects despite their mode of action differences [36]. Perturbation of coregulatory dynamics involving nuclear receptors and histone methylation modification may be a common factor, whereby the target genes being regulated and associated with the compounds' modes of action may differ, but the underlying mechanism, i.e. perturbation of the delicate balance between the actions of histone methyltransferases and demethylases, is the factor compounded by their combinatorial effects. Following exposure, small fold changes in mRNA levels of genes encoding histone modifying enzymes would likely be difficult to detect in studies with small sample numbers. Their role in toxicity modes of action and potential cumulative effects may thus be easily overlooked.

The coexpression patterns observed in testis tissue across multiple biological settings in this study concur with current understanding of nuclear receptor activity, whereby receptors act in concert with multiple sub-unit complexes of proteins. The identification of which combinations of coregulatory networks are specific to one or more nuclear receptors and the generality or specificity of their coactivity in the testis is a knowledge-gap that needs to be addressed. The incorporation of such information into algorithms for the prediction of cumulative effects would likely improve results.

### Future studies

This study was restricted by the availability of appropriate data sets in the public corpus and suitable probes on the associated microarray platforms. Studies targeting appropriate cell lines and tissues and specific endocrine disruptors would likely yield more powerful results. Sufficient replicate samples to allow meaningful correlation analysis may be more attainable as the cost of the associated technology reduces over time. This study does, however, provide proof of concept that profiling genes related to a specific hypothesis across multiple biological settings can identify patterns of transcriptional regulation that cannot be evidenced at a single experiment level. These patterns provide a framework against which endocrine disruption can be better understood and assessed. On a broader level, the application of bioinformatics approaches that integrate both methods and multiple data sources has enormous potential to address the complexities inherent in assessing health risk from potential epigenetic toxicants.

### Conclusions

To the best of our knowledge this is the first study to attempt to assess the generality of coregulatory action between nuclear receptors and genes involved in histone methylation across species and studies, and the susceptibility of their coregulator activity to endocrine disruptors. Our results suggest that the gatekeeper mechanism as described by Garcia-Bassets et al and activity of the ASCOM histone methylation modifying complexes are an integral component of nuclear receptor mediated transcriptional regulation in the testis. Genes implicated in these transcriptional mechanisms show common expression patterns with genes key to multiple endocrine disruptor phenotypes and are thus a likely common factor influencing dysregulation following exposure. Given the tight context specificity of binding partners and target substrates, coexpression patterns of distinct combinations of nuclear receptors, histone demethylases and methyltransferases have potential as biomarkers of both normal function and endocrine disruption that warrants further investigation. Most importantly, this work demonstrates that an integrative, multi-experiment approach is required to gain perspective and further understanding of both natural function and potential perturbation of the epigenetic apparatus.

## Materials and Methods

### Analysis geneset

Genes from three main functional groups were considered to be relevant to our hypothesis: nuclear receptors, histone methylation modifiers and genes associated with endocrine disruptor phenotypes. Nuclear receptors (n = 41) were obtained from a review paper on their nomenclature and function [37]. Histone modifiers associated with demethylation (n = 18), and methylation (n = 45) were obtained from reviews by Cloos [9] and Hublitz [11] and a gene ontology search conducted in June 2011. Genes relevant to nuclear receptor coregulation (n = 7) and to endocrine disruptor phenotypes (n = 28) were selected from a literature review conducted in the Web of Science®, see Table S1 for references and details.

### Gene expression data

Raw data were obtained from the European Bioinformatics Institute (EBI) ArrayExpress repository, accession numbers E-TABM-130, E-GEOD-10919, E-GEOD-25196, E-GEOD-13550, E-GEOD-20952, E-GEOD-10412, E-GEOD-22616, E-GEOD-18211, E-GEOD-6881 and E-GEOD- 29963. Experiments were selected for their relevance to endocrine disruption and availability of unprocessed array files and are summarised in Table S4. Experiment 1, E-TABM-130, provided sufficient sample numbers to conduct coexpression analysis in a tissue relevant to endocrine disruption across three species. Samples selected from this data set included 8 human, 12 mouse and 12 rat seminiferous tubules, total testis, spermatocytes and spermatid cells pooled from several juvenile or adult individuals. The study samples had been hybridised to the Affymetrix GeneChip Human Genome U133 Plus 2.0, Affymetrix GeneChip Mouse Genome 430 2.0 and Affymetrix GeneChip Rat Genome 230 2.0 platforms respectively. Experiment 2, E-GEOD-10919, provided 12 testis samples from a vinclozolin exposure experiment conducted over three generations. Samples had been collected at embryonic day 16 and included 2 controls and 2 vinclozolin exposed for each of F1 through to F3 generations. Experiment 3, E-GEOD-25196, provided samples from controls and rats exposed *in utero* to the known endocrine disruptor dibutyl phthalate daily from gestational day (GD) 12 to 20. Samples from exposed testis had been collected 6 hours following the final dose. Samples from Experiment 4, E-GEOD-13550, were obtained from rats exposed *in utero* to dibutyl phthalate or a control vehicle from gestational day 12 to 20 (as per Experiment 3), but these samples were not collected until post natal day 35. Samples selected from Experiment 5, E-GEOD-20952, included five controls and five samples from rats exposed to a single dose of dibutyl phthalate at 10 week of age. Experiments involving exposure to dibutyl Phthalate were selected because this compound had known endocrine disruptor properties. Experiment 6, E-GEOD-10412 provided samples from a toxicogenomics study involving three triazole compounds. This dataset was selected to gain insight into the profile of the genes of interest following exposure to a group of similar chemicals. Experiment 7, E-GEOD-226616 provided samples from three testis tissues in the mouse (efferent ducts, epididymis and vas deferens) and was selected to allow profiling of the geneset in different tissues of the testis. Mouse embryonic testis tissue from Experiment 8, E-GEOD-18211, and Experiment 9, E-GEOD-18211, were selected to allow comparison between mouse and rat embryonic tissue and adult and embryonic mouse tissue. Experiment 10, E-GEOD-29963 provided samples from specific cell types (spermatocytes and round spermatids) and was selected to compare coexpression patterns in these cells and to compare these results with those observed in whole testis tissue. Of the total 137 genes selected for our enriched geneset, all were available on the mouse microarray platform, 136 were available on human and 122 on the rat microarrays.

### Microarray data normalization

Affymetrix CEL files were downloaded from ArrayExpress and normalised using the robust multiarray average (RMA) algorithm [38] available in the *affy* package of Bioconductor [39]. For each experiment box plots of samples across all probes showed the arrays to be similarly distributed and controls clustered separately from exposed samples. Affymetrix chip annotation databases were used for probe to gene mapping. For reporting purposes, where different synonyms were used for histone demethylases they were converted to the recent nomenclature standard for these genes [40]. For example, the gene symbol *Utx*, used in the human and rat annotation packages, was converted to the new nomenclature *Kdm6a*. Where more than one probe was available for a gene the probe with the highest average expression across normal samples was selected for each biological setting.

### Differential expression analysis

Statistical analyses were carried out using Bioconductor within the R statistical software environment [38]. Differential expression was determined using the *lmFit* and *eBayes* functions available in the *limma* package [41]. The *lmFit* function was used to fit a linear model for each gene in the geneset allowing estimation of the coefficient comparing controls and each compound exposed group. The *eBayes* function was then used to compute moderated t-statistics and p-values for the coefficient estimates from *lmFit*. The p-values were adjusted for multiple testing using the Benjamini & Hochberg method [42]. Genes differentially expressed by a fold change greater than or equal to 1.5 and an adjusted p-value less than 0.05 are reported. A relatively modest fold change cut-off point was selected since the histone modifying genes, the focus of this study, are known to be tightly regulated and small differences are likely to have biological relevance.

### Coexpression analysis

Fundamental to this work was the hypotheses that genes with similar expression profiles were likely to have coactivity or be functionally influenced by each other. In 2004 Allocco et al [43] tested the similar hypothesis that genes with similar expression profiles are likely to be regulated by the same mechanisms and showed that genes with a correlation between their expression profiles above 0.84 have a greater than 50% chance of sharing a common transcription factor binder site. Here, Pearson correlation coefficients (*r*) were calculated and an absolute-value threshold of 0.75, p-value<0.05, was used to detect coexpression patterns between each gene and all others in the analysis set. A lower threshold than that used by Allocco was selected as the coregulatory relationships we aimed to investigate were broader in nature than the more targeted objective of the Allocco study. The samples from experiment 1 were pooled from several cell types and in Experiment 4 samples from three generations were grouped as either exposed or non-exposed. Partial correlation was used to calculate the Pearson correlation for these experiments to take into account the sample and generation groupings. Partial correlation was also applied for the analysis of Experiments 7–9 where samples from different time points were grouped to provide sufficient samples sizes for coexpression analysis.

## Supporting Information

Figure S1 **Ingenutiy Pathway Analysis (IPA) network of ASCOM components.** Network showing interactions between histone modifier proteins: gene identifiers for the geneset were uploaded into the IPA application. Each identifier was mapped to its corresponding object in the Ingenuity® Knowledge Base. A core analysis was conducted that generated networks of molecules based on their connectivity. The most comprehensive of which is shown. Connections between molecules are based on information contained in the Ingenuity Knowledge base and represent at least nine publications reporting molecule interactions in a range of non-testis tissues and cell types.
(PDF)

Table S1 **Analysis geneset.**
(PDF)

Table S2 **Conserved coexpression across species and in experiments in rat.**
(PDF)

Table S3 **Conserved coexpression in mouse.**
(PDF)

Table S4 **Data sources.**
(PDF)

## References

[1] Diamanti-Kandarakis E, Bourguignon J, Giudice LC, Hauser R, Prins G (2009). Endocrine-Disrupting Chemicals: An Endocrine Society Scientific Statement.. Endocrine Reviews.

[2] Foster P, Foster (2006). Disruption of reproductive development in male rat offspring following in utero exposure to phthalate esters.. Int J Androl.

[3] Newbold R, Padilla-Banks E, Jefferson W (2009). Environmental estrogens and obesity.. Mol Cell Endocrinol.

[4] Narita M, Miyagawa K, Mizuo K, Yoshida T, Suzuki T (2007). Changes in central dopaminergic systems and morphine reward by prenatal and neonatal exposure to bisphenol-A in mice: evidence for the importance of exposure period.. Addict Biol.

[5] Wilson VS, Blystone CR, Hotchkiss AK, Rider CV, Gray LE (2008). Diverse mechanisms of anti-androgen action: impact on male rat reproductive tract development.. Int J Androl.

[6] Zhang X, Ho S (2011). Epigenetics meets endocrinology.. Journal of molecular endocrinology.

[7] LeBaron M, Rasoulpour R, Klapacz J, Ellis-Hutchings R, Hollnagel H (2010). Epigenetics and chemical safety assessment.. Mutation research - Reviews in mutation research.

[8] Bjornstrom L, Sjoberg M (2005). Mechanisms of estrogen receptor signaling: Convergence of genomic and nongenomic actions on target genes.. Molecular endocrinology.

[9] Cloos PA, Christensen J, Agger K, Helin K (2008). Erasing the methyl mark: histone demethylases at the center of cellular differentiation and disease.. Genes Dev.

[10] Bulynko Y, O'Malley B (2011). Nuclear Receptor Coactivators: Structural and Functional Biochemistry.. Biochemistry (Mosc).

[11] Hublitz P, Albert M, Peters A (2009). Mechanisms of transcriptional repression by histone lysine methylation.. The international journal of developmental biology.

[12] Hiroi H, Christenson L, Chang L, Sammel M, Berger S (2004). Temporal and spatial changes in transcription factor binding and histone modifications at the steroidogenic acute regulatory protein (StAR) locus associated with StAR transcription.. Molecular endocrinology.

[13] Kim S, Kim J, Choe N, Cho I, Kim D (2010). Regulation of mouse steroidogenesis by WHISTLE and JMJD1C through histone methylation balance.. Nucleic acids research.

[14] Okada Y, Tateishi K, Zhang Y (2010). Histone Demethylase JHDM2A Is Involved in Male Infertility and Obesity.. J Androl.

[15] Lee S, Kim D, Goo Y, Lee Y, Lee J (2009). Crucial Roles for Interactions between MLL3/4 and INI1 in Nuclear Receptor Transactivation.. Molecular endocrinology.

[16] Metzger E, Wissmann M, Yin N, Muller J, Schneider R (2005). LSD1 demethylates repressive histone marks to promote androgen-receptor-dependent transcription.. Nature.

[17] Yang J, Jubb A, Pike L, Buffa F, Turley H (2010). The Histone Demethylase JMJD2B Is Regulated by Estrogen Receptor alpha and Hypoxia, and Is a Key Mediator of Estrogen Induced Growth.. Cancer Res.

[18] Garcia-Bassets I, Kwon Y, Telese F, Prefontaine G, Hutt K (2007). Histone methylation-dependent mechanisms impose ligand dependency for gene activation by nuclear receptors.. Cell.

[19] De Santa F, Totaro M, Prosperini E, Notarbartolo S, Testa G (2007). The histone H3 lysine-27 demethylase Jmjd3 links inflammation to inhibition of polycomb-mediated gene silencing.. Cell.

[20] Issaeva I (2007). Knockdown of ALR (MLL2) Reveals ALR Target Genes and Leads to Alterations in Cell Adhesion and Growth.. Mol Cell Biol.

[21] Lee S, Lee J (2008). Activating signal cointegrator-2 is an essential adaptor to recruit histone H3 lysine 4 methyltransferases MLL3 and MLL4 to the liver X receptors.. Molecular endocrinology.

[22] Mo R, Rao S, Zhu Y (2006). Identification of the MLL2 complex as a coactivator for estrogen receptor alpha.. Journal of Biological Chemistry.

[23] Ansari K, Shrestha B, Hussain I, Kasiri S, Mandal S (2011). Histone Methylases MLL1 and MLL3 Coordinate with Estrogen Receptors in Estrogen-Mediated HOXB9 Expression.. Biochemistry (Mosc).

[24] Dreijerink (2009). The Multiple Endocrine Neoplasia Type 1 (MEN1) Tumor Suppressor Regulates Peroxisome Proliferator-Activated Receptor {gamma}-Dependent Adipocyte Differentiation.. Mol Cell Biol.

[25] Vicent G, Nacht A, Font Mateu J, Castellano G, Gaveglia L (2011). Four enzymes cooperate to displace histone H1 during the first minute of hormonal gene activation.. Genes Dev.

[26] Viswakarma N, Jia Y, Bai L, Vluggens A, Borensztajn J (2010). Coactivators in PPAR-Regulated Gene Expression..

[27] Newbold R (2011). Developmental exposure to endocrine-disrupting chemicals programs for reproductive tract alterations and obesity later in life.(Author abstract) American Journal of Clinical Nutrition.. The American journal of clinical nutrition.

[28] Sato K, Fukata H, Kogo Y, Ohgane J, Shiota K (2009). Neonatal Exposure to Diethylstilbestrol Alters Expression of DNA Methyltransferases and Methylation of Genomic DNA in the Mouse Uterus.. Endocr J.

[29] Crawford B, Hess J (2006). MLL core components give the green light to histone methylation.. ACS chemical biology.

[30] Wysocka J, Swigut T, Milne TA, Dou YL, Zhang X (2005). WDR5 associates with histone H3 methylated at K4 and is essential for H3 K4 methylation and vertebrate development.. Cell.

[31] Dreijerink KMA, Mulder KW, Winkler GS, Hoppener JWM, Lips CJM (2006). Menin links estrogen receptor activation to histone H3K4 trimethylation.. Cancer Res.

[32] Sun L, Huang L, Nguyen P, Bisht KS, Bar-Sela G (2008). DNA methyltransferase 1 and 3B activate BAG-1 expression via recruitment of CTCFL/BORIS and modulation of promoter histone methylation.. Cancer Res.

[33] Wang J, Hevi S, Kurash J, Lei H, Gay F (2009). The lysine demethylase LSD1 (KDM1) is required for maintenance of global DNA methylation.. Nature genetics.

[34] Hemberger M, Dean W, Reik W (2009). Epigenetic dynamics of stem cells and cell lineage commitment: digging Waddington's canal.. Nature Reviews Molecular Cell Biology.

[35] Brykczynska U, Hisano M, Erkek S, Ramos L, Oakeley E (2010). Repressive and active histone methylation mark distinct promoters in human and mouse spermatozoa.. Nature structural & molecular biology.

[36] Hotchkiss A, Rider C, Furr J, Howdeshell K, Blystone C (2010). In utero exposure to an AR antagonist plus an inhibitor of fetal testosterone synthesis induces cumulative effects on F1 male rats.. Reproductive Toxicology.

[37] Germain P, Staels B, Dacquet C, Spedding M, Laudet V (2006). Overview of nomenclature of nuclear receptors.. Pharmacol Rev.

[38] The R project for statistical computing

[39] Gentleman R, Carey V, Bates D, Bolstad B, Dettling M (2004). Bioconductor: open software development for computational biology and bioinformatics.. GenomeBiologycom.

[40] Allis C, Berger S, Cote J, Dent S, Jenuwien T (2007). New nomenclature for chromatin-modifying enzymes.. Cell.

[41] Smyth GK (2004). Linear models and empirical bayes methods for assessing differential expression in microarray experiments.. Stat Appl Genet Mol Biol.

[42] Benjamini Y, Hochberg Y (1995). Controlling the false diiscovery rate - a practical and pwerful approach to multiple testing.. Journal of the Royal Statistical Society Series B Methodological.

[43] Allocco DJ, Kohane IS, Butte AJ (2004). Quantifying the relationship between co-expression, co-regulation and gene function.. BMC Bioinformatics.

