# Supplementary figures and images for: Coexpression of Nuclear Receptors and Histone Methylation Modifying Genes in the Testis: Implications for Endocrine Disruptor Modes of Action
Source: PLoS One. 2012 Apr 4;7(4):e34158. doi: 10.1371/journal.pone.0034158 (PMC3319570; doi:10.1371/journal.pone.0034158)

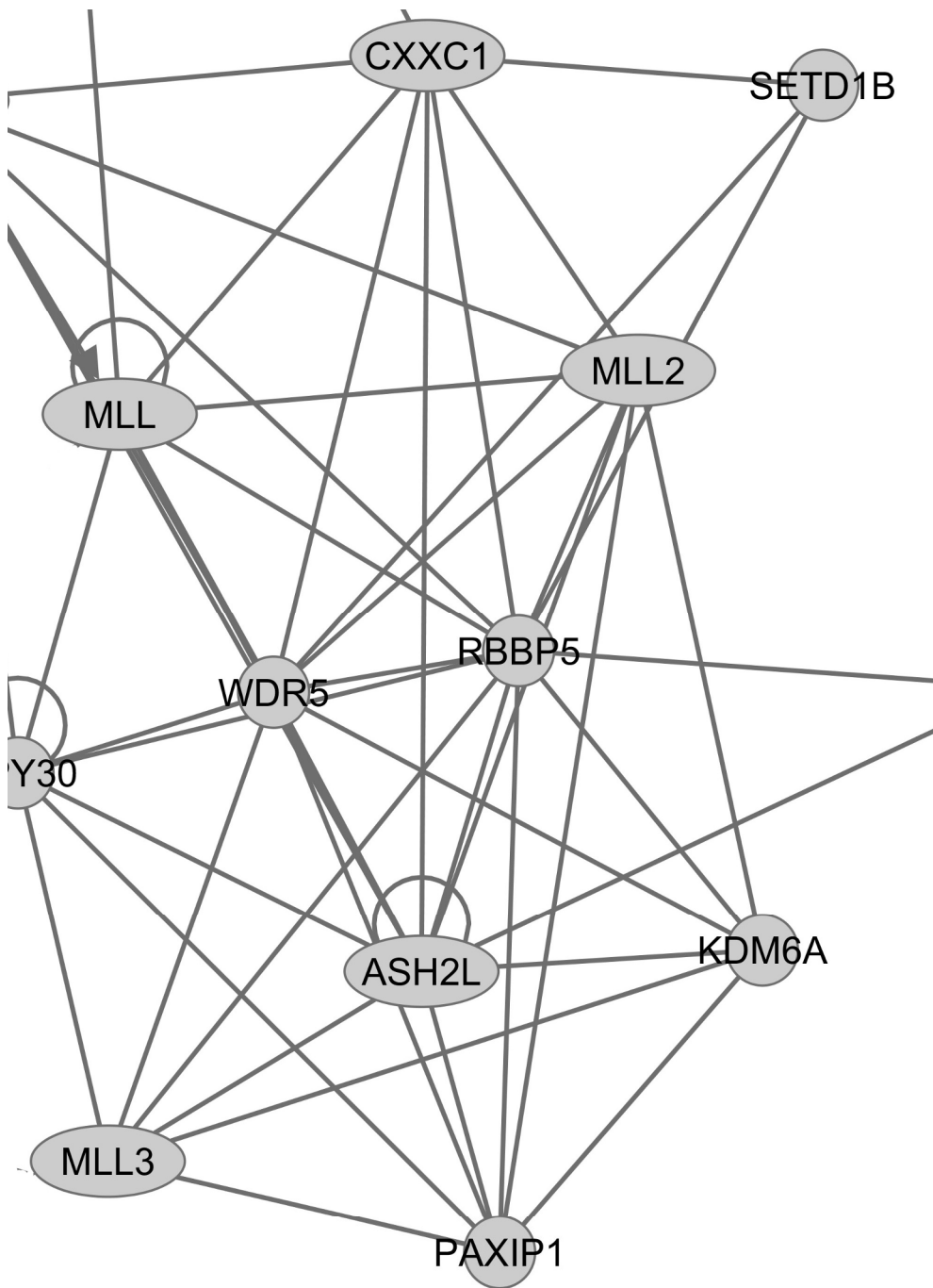

Supplement: Figure S1 — Ingenutiy Pathway Analysis (IPA) network of ASCOM components. Network showing interactions between histone modifier proteins: gene identifiers for the geneset were uploaded into the IPA application. Each identifier was mapped to its corresponding object in the Ingenuity® Knowledge Base. A core analysis was conducted that generated networks of molecules based on their connectivity. The most comprehensive of which is shown. Connections between molecules are based on information contained in the Ingenuity Knowledge base and represent at least nine publications reporting molecule interactions in a range of non-testis tissues and cell types. (PDF) [file pone.0034158.s001.pdf]
