# Supplementary material for: Coexpression of Nuclear Receptors and Histone Methylation Modifying Genes in the Testis: Implications for Endocrine Disruptor Modes of Action
Source: PLoS One. 2012 Apr 4;7(4):e34158. doi: 10.1371/journal.pone.0034158 (PMC3319570; doi:10.1371/journal.pone.0034158)
Supplement: Table S1 — Analysis geneset. (PDF) [file pone.0034158.s002.pdf]

Supplementary Table 1: Analysis gene set								
**Gene Symbol**	**Function**	**Mouse Entrez Gene ID**	**Human Entrez Gene ID**	**Rat Entrez Gene ID**	**Notes/References**	**Human probe ID**	**Mouse probe ID**	**Rat probe ID**
Aof1	Histone demethylase	218214	221656	306819	Substrate: H3K4, Lin, S.L., et al., *Regulation of somatic cell reprogramming through inducible mir-302 expression.* Nucleic Acids Res, 2010	227021_at	1433649_at	1390228_at
Ar	Nuclear receptor	11835	367	24890	Ligands: Testosterone, flutamide	211110_s_at	1455647_at	1369159_at
Ash2l	Histone methylation	23808	9070	290829	Histone H3-K4 methylation GO:0051568	209517_s_at	1420616_at	1388871_at
Baz2a	Histone methylation	116848	11176	304601	Histone H3-K9 methylation GO:0051567	201353_s_at	1438192_s_	1394721_at
Bcor	Histone methylation	71458	54880	317346	Negative regulation of histone H3-K36 methylation GO:0000415,GO:0051572	223566_s_at	1452910_at	1385592_at
Bmp4	EDC phenotype - de	12159	652	25296	Mesoderm cell fate decisions, polycomb group target gene identified by Bracken, A.P., et al., *Genome-wide mapping of Polycomb target genes unravels their roles in cell fate transitions.* Genes Dev, 2006. **20**(9): p. 1123-60	211518_s_at	1422912_at	1387232_at
Bmp7	EDC phenotype - de	12162	655	85272	Sex differentiation, polycomb group target gene identified by Bracken, A.P., et al., *Genome-wide mapping of Polycomb target genes unravels their roles in cell fate transitions.* Genes Dev, 2006. **20**(9): p. 1123-61	211259_s_at	1418910_at	1389403_at
Bmp8a	EDC phenotype - de	12163	353500	680931	Sex differentiation, polycomb group target gene identified by Bracken, A.P., et al., *Genome-wide mapping of Polycomb target genes unravels their roles in cell fate transitions.* Genes Dev, 2006. **20**(9): p. 1123-62	220203_at	1449873_at	1381452_at
Carm1	Histone methylation	59035	10498	363026	Histone H3-R2 methylation GO:0034970	212512_s_at	1419743_s_	1399048_at
Crebbp	Coactivator	12914	1387	54244	Viswakarma, N., et al., *Coactivators in PPAR-Regulated Gene Expression.* PPAR Res, 2010.	202160_at	1459804_at	1382264_at
Ctcf	Histone methylation	13018	10664	83726	Substrate H3: K4, regualtion of histone methylation GO:0031060	202521_at	1449042_at	1370337_at
Cxxc1	Histone methylation	74322	30827	291440	Histone H3-K4 methylation GO:0051568	48580_at	1454106_a_	1388875_at
Cyp11a1	EDC phenotype - ste	13070	1583	29680	Wilson, V.S., et al., *Diverse mechanisms of anti-androgen action: impact on male rat reproductive tract development.* Int J Androl, 2008. **31**(2): p. 178-87	204309_at	1439947_at	1368468_at
Cyp17a1	EDC phenotype - ste	13074	1586	25146	Wilson, V.S., et al., *Diverse mechanisms of anti-androgen action: impact on male rat reproductive tract development.* Int J Androl, 2008. **31**(2): p. 178-87	205502_at	1417017_at	

Dmrt1	EDC phenotype - de	50796	1761	114498	Sex differentiation, polycomb group target gene identified by Bracken, A.P., et al., *Genome-wide mapping of Polycomb target genes unravels their roles in cell fate transitions.* Genes Dev, 2006. **20**(9): p. 1123-61	220493_at	1423582_at	1387597_at
Dmrt2	EDC phenotype - de	226049	10655	309430	Sex differentiation, polycomb group target gene identified by Bracken, A.P., et al., *Genome-wide mapping of Polycomb target genes unravels their roles in cell fate transitions.* Genes Dev, 2006. **20**(9): p. 1123-61	223986_x_a	1426867_at	1384487_at
Dmrt3	EDC phenotype - de	240590	58524	n/a	Sex differentiation, polycomb group target gene identified by Bracken, A.P., et al., *Genome-wide mapping of Polycomb target genes unravels their roles in cell fate transitions.* Genes Dev, 2006. **20**(9): p. 1123-61	231800_s_at	1440707_at	n/a
Dmrta1	EDC phenotype - de	242523	63951	313352	Sex differentiation, polycomb group target gene identified by Bracken, A.P., et al., *Genome-wide mapping of Polycomb target genes unravels their roles in cell fate transitions.* Genes Dev, 2006. **20**(9): p. 1123-61	244725_at	1441579_at	1386239_at
Dnmt1	Histone methylation	13433	1786	84350	Substrates: H3K4,H3K9, negative regulation of histone H3-K9 methylation GO:0051573,GO:0051571	201697_s_at	1447877_x_	1373772_at
Dnmt3b	Histone methylation	13436	1789	444985	Substrates: H3K4,H3K9, positive regulation of histone H3-K4 methylation GO:0051571,GO:0051573	220668_s_at	1449052_a_	1385662_at
Dot1l	Histone methylation	208266	84444	362831	Substrate: H3K79, histone methylation GO:0016571	231297_at	1457268_at	1388589_at
Dpy30	Histone methylation	66310	84661	286897	Histone H3-K4 methylation GO:0051568	224129_s_at	1423767_at	1371982_at
Eed	Histone methylation	13626	8726	293104	Substrates: H3K9,H3K27, histone methylation GO:0016571	209572_s_at	1453830_at	1373462_at
Ehmt1	Histone methylation	77683	79813	362078	Substrate: H3K9 histone methylation GO:0016571	225461_at	1454776_at	1376578_at
Ehmt2	Histone methylation	110147	10919	361798	Histone H3-K9 methylation GO:0051567	202326_at	1460692_at	1388716_at
Ep300	Coactivator	328572	2033	170915	Viswakarma, N., et al., *Coactivators in PPAR-Regulated Gene Expression.* PPAR Res, 2010.	202221_s_at	1434765_at	1373916_at
Esr1	Nuclear receptor	13982	2099	24890	Ligands: Estradiol-17b,tamoxifen,raloxifene	211234_x_a	1421244_at	1387704_at
Esr2	Nuclear receptor	13983	2100	5149	Ligands: Estradiol-17b,various synthetic compounds	211118_x_a	1426103_a_	1369760_a at
Ezh1	Histone methylation	14055	2145	303547	Substrate: H3K27, Hublitz, P., M. Albert, and A. Peters, Mechanisms of transcriptional repression by histone lysine methylation. The international journal of developmental biology, 2009. 53(2-3): p. 335-354	203249_at	1449023_a_	1399059_at
Ezh2	Histone methylation	14056	2146	312299	Substrate H3K27,histone methylation GO:0016571	203358_s_at	1416544_at	1373290_at

Fgf9	EDC phenotype - de	14180	2254	25444	Sex differentiation, polycomb group target gene identified by Bracken, A.P., et al., *Genome-wide mapping of Polycomb target genes unravels their roles in cell fate transitions.* Genes Dev, 2006. **20**(9): p. 1123-61	206404_at	1420795_at	1392865_at
Hdac1	corepressor	n/a	3065	297893	Viswakarma, N., et al., *Coactivators in PPAR-Regulated Gene Expression.* PPAR Res, 2010. **2010**.	201209_at	1448246_at	1396820_at
Hdac2	corepressor	15182	3066	n/a	Viswakarma, N., et al., *Coactivators in PPAR-Regulated Gene Expression.* PPAR Res, 2010. **2010**.	201833_at	1445684_s_	1370908_at
Hoxa10	EDC phenotype - de	15395	3206	368057	Regulation of transcription, DNA-dependent. Development. Spermatogenesis, polycomb group target gene identified by Bracken, A.P., et al., *Genome-wide mapping of Polycomb target genes unravels their roles in cell fate transitions.* Genes Dev, 2006. **20**(9): p. 1123-86	213147_at	1446408_at	1398398_at
Hoxa11	EDC phenotype - de	15396	3207	n/a	Regulation of transcription, Morphogenesis, polycomb group target gene identified by Bracken, A.P., et al., *Genome-wide mapping of Polycomb target genes unravels their roles in cell fate transitions.* Genes Dev, 2006. **20**(9): p. 1123-87	213823_at	1420414_at	n/a
Hoxa4	EDC phenotype - de	15401	3201	n/a	Regulation of transcription, Morphogenesis, polycomb group target gene identified by Bracken, A.P., et al., *Genome-wide mapping of Polycomb target genes unravels their roles in cell fate transitions.* Genes Dev, 2006. **20**(9): p. 1123-88	206289_at	1427354_at	n/a
Hoxc13	EDC phenotype - de	15422	3229	n/a	Regulation of transcription, Morphogenesis polycomb group target gene identified by Bracken, A.P., et al., *Genome-wide mapping of Polycomb target genes unravels their roles in cell fate transitions.* Genes Dev, 2006. **20**(9): p. 1123-89	219832_s_at	1425874_at	n/a
Hoxd3	EDC phenotype - de	15434	3232	288152	Regulation of transcription, Morphogenesis polycomb group target gene identified by Bracken, A.P., et al., *Genome-wide mapping of Polycomb target genes unravels their roles in cell fate transitions.* Genes Dev, 2006. **20**(9): p. 1123-90	206601_s_at	1421537_at	1398314_at
Hoxd8	EDC phenotype - de	15437	3234	NA	Regulation of transcription, Development, Determination of anterior/posterior axis, embryo polycomb group target gene identified by Bracken, A.P., et al., *Genome-wide mapping of Polycomb target genes unravels their roles in cell fate transitions.* Genes Dev, 2006. **20**(9): p. 1123-91	231906_at	1431099_at	n/a

Insl3	EDC-phenotype spe	n/a	n/a	114215	Wilson, V.S., et al., *Diverse mechanisms of anti-androgen action: impact on male rat reproductive tract development.* Int J Androl, 2008. **31**(2): p. 178-87	n/a	n/a	1388241_at
Jag2	EDC phenotype - de	16450	3714	29147	Sex differentiation, polycomb group target gene identified by Bracken, A.P., et al., *Genome-wide mapping of Polycomb target genes unravels their roles in cell fate transitions.* Genes Dev, 2006. **20**(9): p. 1123-61	32137_at	1426430_at	1371006_at
Jmjd1c	Histone demethylase	108829	221037	171120	Synonym:JMJD1C, Substrate: H3K9, Kim, S., et al., *Regulation of mouse steroidogenesis by WHISTLE and JMJD1C through histone methylation balance.* Nucleic acids research, 2010. **38**(19): p. 6389-6403.	224933_s_at	1448049_at	1388169_at
Jmjd6	Histone demethylase	107817	23210	360665	Synonym: JMJD6, Substrates: H3R2,H3R3, Cloos, P.A., et al., *Erasing the methyl mark: histone demethylases at the center of cellular differentiation and disease.* Genes Dev, 2008. **22**(9): p. 1115-40.	212723_at	1454109_a_	1374632_at
Kdm1	Histone demethylase	99982	23028	500569	Synonym:AOF2, Substrates: H3K4,H3K9, Hublitz, P., M. Albert, and A. Peters, *Mechanisms of transcriptional repression by histone lysine methylation.* The international journal of developmental biology, 2009. **53**(2-3): p. 335-354	212348_s_at	1426762_s_	1372007_at
Kdm2a	Histone demethylase	225876	22992	361700	Synonym:FBXL11, Substrates: H3K36,H3K4, Hublitz, P., M. Albert, and A. Peters, *Mechanisms of transcriptional repression by histone lysine methylation.* The international journal of developmental biology, 2009. **53**(2-3): p. 335-354	208988_at	1458449_at	1399154_at
Kdm2b	Histone demethylase	30841	84678	304495	Synonym:FBXL10, Substrates: H3K36,H3K4, Hublitz, P., M. Albert, and A. Peters, *Mechanisms of transcriptional repression by histone lysine methylation.* The international journal of developmental biology, 2009. **53**(2-3): p. 335-354	226215_s_at	1452198_at	1393101_at
Kdm3a	Histone demethylase	104263	55818	312440	Synonym:JMJD1A, Substrate H3K9, Hublitz, P., M. Albert, and A. Peters, *Mechanisms of transcriptional repression by histone lysine methylation.* The international journal of developmental biology, 2009. **53**(2-3): p. 335-354	212689_s_at	1426810_at	1370975_at
Kdm3b	Histone demethylase	277250	51780	n/a	Synonym:JMJD1B, Substrate:H3K9, Hublitz, P., M. Albert, and A. Peters, *Mechanisms of transcriptional repression by histone lysine methylation.* The international journal of developmental biology, 2009. **53**(2-3): p. 335-354	201643_x_a	1458417_at	n/a

Kdm4a	Histone demethylase	230674	9682	n/a	Synonym:JMJD2A, Substrates: H3K36,H3K9, Hublitz, P., M. Albert, and A. Peters, *Mechanisms of transcriptional repression by histone lysine methylation.* The international journal of developmental biology, 2009. **53**(2-3): p. 335-354	203204_s_at	1446699_at	n/a
Kdm4b	Histone demethylase	193796	23030	n/a	Synonym:JMJD2B, Substrates: H3K36,H3K9, Hublitz, P., M. Albert, and A. Peters, *Mechanisms of transcriptional repression by histone lysine methylation.* The international journal of developmental biology, 2009. **53**(2-3): p. 335-354	212496_s_at	1424044_at	n/a
Kdm4c	Histone demethylase	76804	23081	n/a	Synonym:JMJD2C, Substrates: H3K36,H3K9, Hublitz, P., M. Albert, and A. Peters, *Mechanisms of transcriptional repression by histone lysine methylation.* The international journal of developmental biology, 2009. **53**(2-3): p. 335-354	244385_at	1424458_at	n/a
Kdm4d	Histone demethylase	244694	55693	n/a	Synonym:JMJD2D, Substrates: H3K36,H3K9, Cloos, P.A., et al., *Erasing the methyl mark: histone demethylases at the center of cellular differentiation and disease.* Genes Dev, 2008. **22**(9): p. 1115-40.	220278_at	1440105_at	n/a
Kdm5a	Histone demethylase	214899	5927	24710	Synonym:JARID1A,Rbp2, Substrate:H3K4, Hublitz, P., M. Albert, and A. Peters, *Mechanisms of transcriptional repression by histone lysine methylation.* The international journal of developmental biology, 2009. **53**(2-3): p. 335-354	202040_s_at	1452360_a_	1387766_a_at
Kdm5b	Histone demethylase	75605	10765	304809	Synonym:JARID1B, Substrate:H3K4, Hublitz, P., M. Albert, and A. Peters, *Mechanisms of transcriptional repression by histone lysine methylation.* The international journal of developmental biology, 2009. **53**(2-3): p. 335-354	201548_s_at	1457482_at	1376347_at
Kdm5c	Histone demethylase	20591	8242	n/a	Synonym:JARID1C, Substrate:H3K4, Hublitz, P., M. Albert, and A. Peters, *Mechanisms of transcriptional repression by histone lysine methylation.* The international journal of developmental biology, 2009. **53**(2-3): p. 335-354	202383_at	1457930_at	n/a
Kdm5d	Histone demethylase	20592	8284	n/a	Synonym:JARID1D, Substrate:H3K4, Hublitz, P., M. Albert, and A. Peters, *Mechanisms of transcriptional repression by histone lysine methylation.* The international journal of developmental biology, 2009. **53**(2-3): p. 335-354	206700_s_at	1452563_a_	n/a

Kdm6a	Histone demethylase	22289	7403	317178	Synonym:UTX, Substrate:H3K27, Hublitz, P., M. Albert, and A. Peters, *Mechanisms of transcriptional repression by histone lysine methylation.* The international journal of developmental biology, 2009. **53**(2-3): p. 335-354	203992_s_at	1445198_at	1383720_at
Kdm6b	Histone demethylase	216850	23135	363630	Synonym:JMJD3, Substrate:H3K27, Hublitz, P., M. Albert, and A. Peters, *Mechanisms of transcriptional repression by histone lysine methylation.* The international journal of developmental biology, 2009. **53**(2-3): p. 335-354	1556067_a_	1456610_at	1390000_at
Lep	EDC phenotype - ob	16846	3952	25608	Newbold, R., et al., *Developmental exposure to endocrine disru*	211356_x_a	1425875_a_	1388725_at
Lepr	EDC phenotype - ob	16847	3953	24536	Newbold, R., et al., *Developmental exposure to endocrine disru*	207092_at	1422582_at	1387748_at
Leprot	EDC phenotype - ob	230514	54741	56766	Newbold, R., et al., *Developmental exposure to endocrine disru*	220750_s_at	1421462_a_	1367967_at
Mecp2	Histone methylation	17257	4204	29386	Substrate:H3K9, histone methylation GO:0016571,GO:0031061	202616_s_at	1460246_at	1369277_at
Men1	Histone methylation	17283	4221	29417	Substrate:H3K4, positive regulation of histone methylation GO:0031062	202645_s_at	1443826_x_	1398785_at
Mll1	Histone methylation	214162	n/a	315606	Histone H3-K4 methylation GO:0051568,GO:0051569	na	1452377_at	1375793_at
Mll2	Histone methylation	381022	8085	362996	Subtrate:H3K4, Hublitz, P., M. Albert, and A. Peters, Mechanisms of transcriptional repression by histone lysine methylation. The international journal of developmental biology, 2009. 53(2-3): p. 335-354	231974_at	1427555_at	1394905_at
Mll3	Histone methylation	231051	58508	n/a	Substrate:H3K4, Hublitz, P., M. Albert, and A. Peters, Mechanisms of transcriptional repression by histone lysine methylation. The international journal of developmental biology, 2009. 53(2-3): p. 335-354	222413_s_at	1457193_at	n/a
Mll4	Histone methylation	n/a	n/a	361543	Substrate:H3K4, histone H3-K4 methylation GO:0051568	n/a	n/a	1388951_at
Mll5	Histone methylation	69188	55904	311968	Substrate:H3K4, histone methylation GO:0016571	223190_s_at	1439108_at	1384125_at
Ncoa6	Coregulator	56406	23054	116464	Viswakarma, N., et al., *Coactivators in PPAR-Regulated Gene Expression.* PPAR Res, 2010.	208979_at	1423374_at	1389028_at
Ncor2	Corepressor	20602	9612	360801	Viswakarma, N., et al., *Coactivators in PPAR-Regulated Gene Expression.* PPAR Res, 2010.	207760_s_at	1448893_at	1372196_at
Nr0b1	Nuclear receptor	11614	190	58850	Orphan receptor	206645_s_at	1417760_at	1369478_at
Nr0b2	Nuclear receptor	23957	8431	117274	Orphan receptor	206410_at	1449854_at	1368376_at
Nr1d1	Nuclear receptor	217166	9572	252917	Orphan receptor	31637_s_at	1426464_at	1370816_at
Nr1d2	Nuclear receptor	353187	9975	259241	Orphan receptor	225768_at	1416958_at	1370541_at
Nr1h2	Nuclear receptor	22260	7376	58851	Ligands: T9001317, GW3965	218215_s_at	1416353_at	1389854_at
Nr1h3	Nuclear receptor	22259	10062	58852	Ligands: Oxysterols, T9001317, GW3965	203920_at	1450444_a_	1387365_at
Nr1h4	Nuclear receptor	20186	9971	60351	Ligands: Bile acids, fexaramine	206340_at	1419105_at	1369073_at

Nr1i2	Nuclear receptor	18171	8856	84835	Ligands: Xenobiotics, 16a-cyanopregnenolone	207203_s_at	1425723_at	1369270_at
Nr1i3	Nuclear receptor	12355	9970	65035	Ligands: Xenobiotics, phenobarbital	207007_at	1425392_a_	1368797_at
Nr2c1	Nuclear receptor	22025	7181	252924	Orphan receptor	204791_at	1418605_at	1394657_at
Nr2c2	Nuclear receptor	22026	7182	50659	Orphan receptor	206038_s_at	1451569_at	1369243_at
Nr2e3	Nuclear receptor	23958	10002	n/a	Orphan receptor	208385_at	1423631_at	n/a
Nr2f1	Nuclear receptor	n/a	n/a	81808	Orphan receptor	n/a	n/a	1387571_at
Nr2f2	Nuclear receptor	11819	7026	113984	Orphan receptor	209121_x_a	1416159_at	1395863_at
Nr2f6	Nuclear receptor	13864	2063	245980	Orphan receptor	209262_s_at	1460648_at	1398826_s at
Nr3c1	Nuclear receptor	14815	2908	24413	Ligands: Cortisol, desamethasone, RU486	201865_x_a	1421867_at	1368222_at
Nr3c2	Nuclear receptor	110784	4306	25672	Ligands: Aldosterone, spirolactone	205259_at	1435991_at	1368476_at
Nr4a1	Nuclear receptor	15370	3164	79240	Orphan receptor	202340_x_a	1416505_at	1386935_at
Nr4a2	Nuclear receptor	18227	4929	54278	Orphan receptor	204621_s_at	1447863_s_	1387410_at
Nr4a3	Nuclear receptor	18124	8013	58853	Orphan receptor	209959_at	1421080_at	1393389_at
Nr5a1	Nuclear receptor	26423	2516	83826	Orphan receptor	210333_at	1418315_at	1393286_at
Nr5a2	Nuclear receptor	26424	2494	60349	Orphan receptor	208337_s_at	1420410_at	1387573_a at
Nr6a1	Nuclear receptor	14536	2649	n/a	Orphan receptor	207742_s_at	1428826_at	n/a
Nsd1	Histone methylation	18193	64324	306764	H3-K36 methylation GO:0010452,GO:0034770	219084_at	1456916_at	1398956_at
Paxip1	Histone methylation	55982	22976	311944	Substrates:H3K36,H3K4, GO:0051568,GO:0051571,GO:000416	212825_at	1454943_a_	1376598_at
Pgr	Nuclear receptor	18667	5241	25154	Ligands: Progesterone, medroxyprogesterone acetate, RU486	208305_at	1439527_at	1387563_at
Ppara	Nuclear receptor	19013	5465	25747	Ligands: Fatty acids,leukotriene B, fibrates	244689_at	1449051_at	1387278_at
Ppard	Nuclear receptor	19015	5467	25682	Ligands: Fatty acids	37152_at	1439797_at	1387684_at
Pparg	Nuclear receptor	19016	5468	25664	Ligands: Fatty acids, prostaglandin J, thiazolidinediones	208510_s_at	1420715_a_	1369179_a at
Prdm2	Histone methylation	110593	7799	313678	Hublitz, P., M. Albert, and A. Peters, Mechanisms of transcriptional repression by histone lysine methylation. The international journal of developmental biology, 2009. 53(2-3): p. 335-354 .	203057_s_at	1453068_at	1370639_at
Prdm5	Histone methylation	70779	11107	689788	Substrate:H3K9, histone H3-K9 methylation GO:0051567	220792_at	1442740_at	1384311_at
Prdm9	Histone methylation	213389	56979	n/a	Histone H3-K4 methylation GO:0051568,GO:0034770	221151_at	1426107_at	na
Prm1	Phenotype - spermat	19118	5619	24685	Okada, Y., et al., *Histone demethylase JHDM2A is critical for Tnp1 and Prm1 transcription and spermatogenesis.* Nature, 2007. **450**(7166): p. 119.	206358_at	1415955_x_	1394007_at

Prm2	Phenotype - spermat	19119	5620	25345	Okada, Y., et al., *Histone demethylase JHDM2A is critical for Tnp1 and Prm1 transcription and spermatogenesis.* Nature, 2007. **450**(7166): p. 119.	210122_at	1448105_at	1370039_at
Prmt6	Histone methylation	99890	55170	295384	Histone H3-R2 methylation GO:0034970	223275_at	1457071_x_	1373910_at
Prmt7	Histone methylation	214572	54496	361402	Substrate:H4R3, histone arginine methylation GO:0034969,GO:0043985	219408_at	1451248_at	1382461_at
Prmt8	Histone methylation	381813	56341	n/a	Substrate: unknown, histone arginine methylation, histone methylation GO:0034969, GO:0016571	230839_at	1435204_at	na
Rara	Nuclear receptor	19401	5914	24705	Ligand: Retinoic acid	203749_s_at	1450180_a_	1373579_at
Rarb	Nuclear receptor	218772	5915	24706	Ligand: Retinoic acid	208412_s_at	1454906_at	1376755_at
Rarg	Nuclear receptor	19411	5916	685072	Ligand: Retinoic acid	204189_at	1419415_a_	1390771_at
Rbbp5	Histone methylation	213464	5929	304794	Histone H3-K4 methylation GO:0051568	205169_at	1428886_at	1381323_at
Rora	Nuclear receptor	19883	6095	300807	Ligands: Cholesterol, cholesteryl sulfate	210426_x_a	1424034_at	1379397_at
Rorb	Nuclear receptor	225998	6096	309288	Ligand: Retinoic acid	206443_at	1425162_at	1371257_at
Rorc	Nuclear receptor	19885	6097	368158	Retinoic acid signaling	228806_at	1425792_a_	1379833_at
Rxra	Nuclear receptor	20181	6256	25271	Ligand: Retinoic acid	202449_s_at	1454773_at	1371668_at
Rxrb	Nuclear receptor	20182	6257	361801	Ligand: Retinoic acid	209148_at	1416990_at	1389051_at
Rxrg	Nuclear receptor	20183	6258	83574	Ligand: Retinoic acid	205954_at	1418782_at	1392271_at
Satb1	Histone methylation	20230	6304	316164	Substrate:H3K9, histone methylation GO:0016571	203408_s_at	1456902_at	1378629_at
Set1b	Histone methylation	208043		n/a	Substrate:H3K4, Hublitz, P., M. Albert, and A. Peters, Mechanisms of transcriptional repression by histone lysine methylation. The international journal of developmental biology, 2009. 53(2-3): p. 335-354	213153_at	1437353_at	n/a
Setd2	Histone methylation	235626	29072	316013	Substrate:H3K36, H3-K36 methylation GO:0010452	212493_s_at	1444006_at	1372376_at
Setd8	Histone methylation	67956	387893	689820	Substrate:H4K20, Hublitz, P., M. Albert, and A. Peters, Mechanisms of transcriptional repression by histone lysine methylation. The international journal of developmental biology, 2009. 53(2-3): p. 335-354	228443_s_at	1460687_at	1372458_at
Setdb1	Histone methylation	84505	9869	689883	Substrate:H3K9, Hublitz, P., M. Albert, and A. Peters, Mechanisms of transcriptional repression by histone lysine methylation. The international journal of developmental biology, 2009. 53(2-3): p. 335-354	203155_at	1451833_a_	1382141_at
Shh	EDC phenotype - se:	20423	6469	29499	Mesoderm cell fate decisions, polycomb group target gene identified by Bracken, A.P., et al., *Genome-wide mapping of Polycomb target genes unravels their roles in cell fate transitions.* Genes Dev, 2006. **20**(9): p. 1123-110	236263_at	1436869_at	1387584_at

Sin3a	Corepressor	20466	25942	363067	Viswakarma, N., et al., *Coactivators in PPAR-Regulated Gene Expression.* PPAR Res, 2010.	225135_at	1449343_s_	1392269_at
Smyd1	Histone methylation	12180	150572	297333	Substrate:H3K4, Hublitz, P., M. Albert, and A. Peters, Mechanisms of transcriptional repression by histone lysine methylation. The international journal of developmental biology, 2009. 53(2-3): p. 335-354	243045_at	1450203_at	1397479_at
Smyd2	Histone methylation	226830	56950	289372	Substrate:H3K36, Hublitz, P., M. Albert, and A. Peters, Mechanisms of transcriptional repression by histone lysine methylation. The international journal of developmental biology, 2009. 53(2-3): p. 335-355	212922_s_at	1424760_a_	1371904_at
Smyd3	Histone methylation	69726	64754	498295	Substrate:H3K4, Hublitz, P., M. Albert, and A. Peters, Mechanisms of transcriptional repression by histone lysine methylation. The international journal of developmental biology, 2009. 53(2-3): p. 335-356	218788_s_at	1452817_at	1391046_at
Sry	EDC phenotype - se	21674	6736	25221	Transcription. Regulation of transcription, Sex differentiation, Male sex determination, polycomb group target gene identified by Bracken, A.P., et al., *Genome-wide mapping of Polycomb target genes unravels their roles in cell fate transitions.* Genes Dev, 2006. **20**(9): p. 1123-114	207893_at	1450579_x_	1388279_at
Star	EDC phenotype - ste	20845	6770	25557	Wilson, V.S., et al., *Diverse mechanisms of anti-androgen action: impact on male rat reproductive tract development.* Int J Androl, 2008. **31**(2): p. 178-87	204548_at	1418729_at	1368406_at
Suv39h1	Histone methylation	20937	6839	302553	Histone H3-K9 methylation GO:0051567,GO:0034968	218619_s_at	1432236_a_	1375981_a at
Suv39h2	Histone methylation	64707	79723	364785	Histone H3-K9 methylation GO:0051567,GO:0034968	1554572_a_	1436561_at	1382830_at
Suv420h1	Histone methylation	225888	51111	361688	Histone methylation GO:0016571,GO:0034770	218242_s_at	1455171_at	1390198_at
Suv420h2	Histone methylation	232811	84787	308345	Histone methylation GO:0016571,GO:0034770	224431_s_at	1424059_at	1389620_at
Thra	Nuclear receptor	21833		7067	Ligand: thyroid hormones	35846_at	1454675_at	1380518_s at
Thrb	Nuclear receptor	21834		7068	Ligand: thyroid hormones	207044_at	1422202_at	1387983_at
Ucp1	Phenotype - spermat	22227	7350	24860	Okada, Y., et al., *Histone demethylase JHDM2A is critical for Tnp1 and Prm1 transcription and spermatogenesis.* Nature, 2007. **450**(7166): p. 119	221384_at	1418197_at	1387033_at
Vdr	Nuclear receptor	22337		7421	Ligands: Vitamin D, 1,25-dihydroxyvitamin D	204253_s_at	1418175_at	1369454_at
Wdr5	Histone methylation	140858	11091	362093	Histone H3-K4 methylation GO:0051568	223308_s_at	1448389_at	1388897_at
Whsc1	Histone methylation	107823	7468	680537	Substrate:H3K36, histone lysine methylation GO:0034968	209053_s_at	1455228_at	1391161_at
Whsc1l1	Histone methylation	234135	54904	290831	Substrates: H3K27,H3K4, histone methylation GO:0016571	222544_s_at	1459907_a_	1385941_at

n/a = not available				
**Gene set summary**				
**Function**		**Human**	**Mouse**	**Rat**
Coactivator/corepressor		7	7	7
EDC phenotype		26	26	22
Histone demethylase		18	18	11
Histone methylation		44	45	42
Nuclear receptor		41	41	40
		**136**	**137**	**122**
