# Supplementary material for: Coexpression of Nuclear Receptors and Histone Methylation Modifying Genes in the Testis: Implications for Endocrine Disruptor Modes of Action
Source: PLoS One. 2012 Apr 4;7(4):e34158. doi: 10.1371/journal.pone.0034158 (PMC3319570; doi:10.1371/journal.pone.0034158)
Supplement: Table S2 — Conserved coexpression across species and in experiments in rat. (PDF) [file pone.0034158.s003.pdf]

Supplementary Table 2: Co-expression Relationships Conserved Across Species and Studies					
**A. Conserved across human, rat and mouse testis**					
		**Human**	**Mouse**	**Rat**	
		Pearson (r) p < 0.05	Pearson (r) p < 0.05	Pearson (r) p < 0.05	
**Nuclear receptor**	**Methylation gene**				
Esr1	Ctcf	-0.95	0.89	-0.90	*
Esr1	Dnmt1	-0.90	0.82	-0.90	*
Esr1	Ehmt1	-0.86	0.86	-0.84	*
Esr1	Satb1	-0.94	-0.77	-0.78	
Esr1	Smyd1	0.88	-0.83	0.94	*
Nr1d2	Baz2a (H3K9)	-0.84	-0.75	-0.82	
Nr1d2	Ctcf	0.97	0.79	0.99	
Nr1d2	Dnmt1	0.79	0.87	0.97	
Nr1d2	Ehmt1 (H3K9)	0.79	0.92	0.95	
Nr1d2	Men1	0.96	-0.80	0.90	*
Nr1d2	Satb1	0.95	-0.94	0.80	*
Nr1d2	Setd8 (H4K20, H3K9)	0.81	0.80	0.96	
Nr1d2	Smyd1	-0.85	-0.84	-0.98	
Nr1d2	Smyd3	-0.83	0.86	-0.83	*
Nr1h2	Dpy30	0.88	-0.95	-0.82	*
Nr1h2	Ezh1	0.76	0.76	0.91	
Nr1i2	Setd8	-0.89	-0.88	-0.81	
Nr1i2	Smyd1	0.85	0.88	0.85	
Nr3c1	Ash2l	0.81	-0.84	-0.96	*
Nr3c1	Baz2a	-0.92	-0.96	-0.85	
Nr3c1	Eed	0.97	-0.99	0.84	*
Nr3c1	Ehmt1	0.83	0.99	0.97	
Nr3c1	Satb1	0.99	-0.84	0.89	*
Nr3c1	Setd8	0.93	0.92	0.94	
Nr3c1	Smyd1	-0.82	-0.96	-0.94	
Nr5a1	Dnmt1	-0.80	-0.81	0.85	*
Nr5a1	Rbbp5	-0.89	-0.80	0.98	*
Pparg	Smyd1	-0.86	0.89	0.77	*
Rara	Smyd3	0.78	0.76	-0.84	*

Rxra	Suv39h1	0.78	0.88	0.92							
		**Human**	**Mouse**	**Rat**							
		Pearson (r) p < 0.05	Pearson (r) p < 0.05	Pearson (r) p < 0.05							
Rxrg	Dnmt1	-0.83	-0.79	-0.88							
Rxrg	Prdm2	-0.86	-0.82	0.92	*						
Vdr	Ezh1	-0.82	0.91	-0.85	*						
Vdr	Men1	-0.85	-0.77	-0.86							
**Nuclear receptor**	**Demethylation gene**										
Esr1	Kdm2a (H3K4)	-0.94	-0.80	-0.93							
Esr1	Kdm6a	-0.80	0.84	-0.80	*						
Nr1d2	Aof1 (H3K4)	0.99	0.90	0.91							
Nr3c1	Kdm6a (H3K27)	0.84	0.82	0.98							
Nr3c1	Aof1	0.87	0.86	0.97							
Thra	Kdm6a	0.84	0.90	0.90							
**B. Coexpression realtionshipsbetween nuclear receptors and histone modifiers observed in three or more studies in rat**											
**Nuclear receptor**	**Methylation gene**	**Exp 1 adult rat**	**Exp 2 ED 16**	**Exp 3 gdc20**	**Exp 4 pnd20**	**Exp 5 10wks**	**Exp6 adult**	**Exp10 young pachytene (4 months)**	**Exp 10aged pachytene (18 months)**	**Exp 10 young round spermatid (4 months)**	**Exp10 aged round spermatid (18 months)**
		Pearson (r) p < 0.05	Pearson (r) p < 0.05	Pearson (r) p < 0.05	Pearson (r) p < 0.05	Pearson (r) p < 0.05	Pearson (r) p < 0.05	Pearson (r) p < 0.05	Pearson (r) p < 0.05	Pearson (r) p < 0.05	Pearson (r) p < 0.05
Esr1	Cxx1							-0.99		-0.96	-0.90
Esr1	Kdm1				-0.96			-0.92			-0.87
Esr1	Kdm2b							-0.88		-0.88	-0.92
Esr1	Kdm3a							-0.99		-0.98	-0.88
Esr2	Cxx1		-0.83				-0.87			-0.81	
Esr2	Prdm2	0.81	0.95				0.76				

Esr2	Prdm5					-0.91		-0.85		-0.83	
Nr0b1	Nsd1	0.79					0.79	0.93			
Nr0b1	Prdm2					-0.96	-0.83			-0.95	-0.91
Nr0b1	Rbbp5	0.86				0.90	0.76				
Nr0b1	Whsc1				-0.97	-0.98		-0.88			
Nr1d1	Dnmt3b			-0.95				-0.83		-0.96	
Nr1d1	Ehmt2		0.93				0.85		0.82		
Nr1d2	Kdm1	0.89					0.81		0.84		
Nr1d2	Nsd1				0.97		0.83	0.92		0.91	
Nr1d2	Smyd3	-0.83			-0.98			-0.93			
Nr1h3	Men1	-0.83					-0.81	-0.97			
Nr1h3	Nsd1	-0.88						-0.94		-0.84	
Nr1h3	Smyd3		0.91				0.93	0.87			
Nr1h4	Setd8	-0.82		-0.95					-0.84		
Nr1h4	Setdb1	-0.85						-0.85	-0.83		
Nr1i2	Dpy30	0.88						0.85			0.83
Nr1i2	Kdm1	-0.83						-0.88		-0.92	
Nr1i2	Mll5	-0.87	-0.86					-0.95			
Nr1i2	Setdb1	-0.84					-0.95	-0.90			
Nr2c1	Cxxc1						0.97	0.90	0.97	0.95	
Nr2c1	Dnmt1		-0.89					-0.90		-0.94	
Nr2c1	Dot1l						0.85	0.86		0.97	0.87
Nr2c1	Ehmt2	0.80					0.93				0.83
Nr2c1	Kdm2b		0.90				0.80	0.98		0.96	
Nr2c1	Kdm3a						0.98	0.85		0.93	0.92
Nr2c1	Whsc1				0.96			0.89		0.93	0.91
Nr2f2	Whsc1	0.82							0.83	0.87	
Nr2f6	Cxxc1			-0.97				-0.93			-0.85
Nr3c1	Bcor	0.76	0.83				0.80				0.82
Nr3c1	Carm1	0.79			0.99						0.90
Nr3c1	Eed	0.84					0.84		0.86		
Nr3c1	Jmjd1c			0.97			0.83		0.82		
Nr3c1	Kdm1	0.88			0.96			0.83			

Nr4a1	Bcor		0.88					0.87			0.87
Nr4a1	Ezh2		0.86							0.87	0.92
Nr4a1	Suv39h2							0.88		0.88	0.95
Nr4a2	Dnmt1						0.87	0.81		0.96	
Nr4a2	Eed	0.79	0.94	0.95				0.97			
Nr4a2	Kdm3a				-0.98			-0.90		-0.87	
Nr4a2	Kdm6a			0.95			0.84			0.95	
Nr4a2	Satb1	0.83					0.93			0.82	
Nr5a1	Ash2l	-0.83						-0.93	-0.90		
Nr5a2	Setdb1		-0.86	-0.98	-0.95						
Ppard	Ctcf		0.86						0.89	0.92	
Rara	Ezh1		-0.86		-0.96		-0.76				
Rara	Kdm2a		-0.96				-0.90	-0.85			
Rara	Kdm3a	-0.83						-0.84			-0.87
Rorc	Bcor							0.87		0.85	0.96
Rorc	Dpy30	0.86						0.99	0.82		0.82
Rorc	Paxip1							0.97		0.94	0.94
Rorc	Suv39h2	0.87						0.92	0.86	0.85	
Rxra	Setdb1			0.97	0.96		0.93				
Rxrg	Dnmt1	-0.88			-0.97						-0.90
Thra	Mll2						0.87			0.93	0.88
Vdr	Wdr5	-0.81			-0.97					-0.87	

