# Supplementary material for: Coexpression of Nuclear Receptors and Histone Methylation Modifying Genes in the Testis: Implications for Endocrine Disruptor Modes of Action
Source: PLoS One. 2012 Apr 4;7(4):e34158. doi: 10.1371/journal.pone.0034158 (PMC3319570; doi:10.1371/journal.pone.0034158)
Supplement: Table S3 — Conserved coexpression in mouse. (PDF) [file pone.0034158.s004.pdf]

Supplementary Table 3: Coexpression Relationships Conserved Across Mouse Tissue

A. Conserved coexpression across mouse epididyms, vas deferens and efferent ducts

Nuclear Receptor	Histone Methylation modifier	Pearson (r) $p < 0.05$	Tissue
Ar	Kdm3a	0.83	effduct
Ar	Kdm3a	0.83	epididymus
Esr1	Ehmt2	-0.77	vasdef
Esr1	Ehmt2	-0.88	epididymus
Esr1	Prmt7	-0.87	epididymus
Esr1	Prmt7	-0.93	vasdef
Esr1	Prmt8	-0.84	epididymus
Esr1	Prmt8	-0.79	vasdef
Esr1	Suv420h2	-0.83	epididymus
Esr1	Suv420h2	-0.83	vasdef
Nr1d1	Smyd3	0.82	epididymus
Nr1d1	Smyd3	0.77	vasdef
Nr1d2	Jmjd1c	-0.88	effduct
Nr1d2	Jmjd1c	-0.86	epididymus
Nr1h2	Carm1	0.88	epididymus
Nr1h2	Carm1	0.83	vasdef
Nr1h2	Ehmt1	0.76	epididymus
Nr1h2	Ehmt1	0.77	vasdef
Nr1h2	Ehmt2	0.88	epididymus
Nr1h2	Ehmt2	0.88	vasdef
Nr1h2	Ezh2	0.85	vasdef
Nr1h2	Ezh2	0.95	epididymus
Nr1h2	Suv420h2	0.96	epididymus
Nr1h2	Suv420h2	0.79	effduct
Nr1h2	Suv420h2	0.93	vasdef
Nr1i3	Satb1	0.78	vasdef

Nr1i3	Satb1	0.75	effduct					
Nr2c1	Ehmt2	0.83	epididymus					
Nr2c1	Ehmt2	0.84	vasdef					
Nr2c1	Smyd2	0.80	epididymus					
Nr2c1	Smyd2	0.80	vasdef					
Nr2c2	Nsd1	-0.78	effduct					
Nr2c2	Nsd1	-0.77	vasdef					
Nr2e3	Ctcf	0.75	epididymus					
Nr2e3	Ctcf	0.90	effduct					
Nr2e3	Kdm5a	0.85	epididymus					
Nr2e3	Kdm5a	0.78	effduct					
Nr3c2	Ehmt2	-0.81	effduct					
Nr3c2	Ehmt2	-0.80	epididymus					
Nr4a2	Dot1l	-0.83	vasdef					
Nr4a2	Dot1l	-0.83	epididymus					
Nr4a2	Ehmt1	-0.83	vasdef					
Nr4a2	Ehmt1	-0.82	epididymus					
Nr4a2	Kdm2b	-0.80	vasdef					
Nr4a2	Kdm2b	-0.88	epididymus					
Nr4a2	Satb1	-0.95	vasdef					
Nr4a2	Satb1	-0.92	epididymus					
Nr4a3	Smyd3	-0.78	vasdef					
Nr4a3	Smyd3	-0.84	epididymus					
Nr6a1	Ezh2	0.85	vasdef					
Nr6a1	Ezh2	0.84	epididymus					
Ppara	Bcor	-0.80	effduct					
Ppara	Bcor	-0.77	epididymus					
Ppard	Ehmt1	0.87	epididymus					
Ppard	Ehmt1	0.86	vasdef					
Ppard	Ehmt2	0.85	epididymus					
Ppard	Ehmt2	0.96	vasdef					
Ppard	Ezh2	0.89	epididymus					
Ppard	Ezh2	0.90	vasdef					

Ppard	Prmt7	0.78	vasdef					
Ppard	Prmt7	0.81	epididymus					
Ppard	Prmt8	0.88	vasdef					
Ppard	Prmt8	0.85	epididymus					
Ppard	Suv420h2	0.89	epididymus					
Ppard	Suv420h2	0.90	vasdef					
Ppard	Bcor	-0.77	vasdef					
Ppard	Bcor	-0.79	epididymus					
Ppard	Mecp2	-0.82	epididymus					
Ppard	Mecp2	-0.81	vasdef					
Rara	Cxxc1	0.88	vasdef					
Rara	Cxxc1	0.80	epididymus					
Rara	Eed	-0.87	epididymus					
Rara	Eed	-0.83	effduct					
Rara	Prdm5	-0.90	epididymus					
Rara	Prdm5	-0.91	vasdef					
Rarb	Suv39h1	0.87	effduct					
Rarb	Suv39h1	0.93	vasdef					
Rarb	Suv39h1	0.78	epididymus					
Rarg	Aof1	0.75	epididymus					
Rarg	Aof1	0.91	vasdef					
Rarg	Ehmt2	0.95	vasdef					
Rarg	Ehmt2	0.85	epididymus					
Rarg	Prmt7	0.91	vasdef					
Rarg	Prmt7	0.96	epididymus					
Rarg	Bcor	-0.82	vasdef					
Rarg	Bcor	-0.78	epididymus					
Rxra	Ehmt2	0.75	effduct					
Rxra	Ehmt2	0.78	epididymus					
Rxra	Satb1	0.78	epididymus					
Rxra	Satb1	0.76	vasdef					
Rxrb	Carm1	0.83	epididymus					
Rxrb	Carm1	0.75	vasdef					

Rxrb	Paxip1	0.79	vasdef	
Rxrb	Paxip1	0.87	effduct	
Rxrb	Jmjd6	-0.84	epididymus	
Rxrb	Jmjd6	-0.84	vasdef	
Rxrg	Kdm2b	0.85	effduct	
Rxrg	Kdm2b	0.89	epididymus	
Rxrg	Kdm2b	0.83	vasdef	
Rxrg	Mll1	-0.80	vasdef	
Rxrg	Mll1	-0.89	epididymus	
Rxrg	Nsd1	-0.93	epididymus	
Rxrg	Nsd1	-0.95	vasdef	
Thra	Dnmt3b	0.88	vasdef	
Thra	Dnmt3b	0.79	epididymus	

B. Conserved coexpression across embryonic mouse tissue

Nuclear Receptor	Histone Methylation Modifier	Pearson (r) p < 0.05	Tissue	Study
Ar	Kdm3a	0.83	effduct	G-EOD-22616
Ar	Kdm3a	0.83	epididymus	G-EOD-22616
Esr1	Ehmt2	-0.77	vasdef	G-EOD-22616
Esr1	Ehmt2	-0.88	epididymus	G-EOD-22616
Esr1	Prmt7	-0.87	epididymus	G-EOD-22616
Esr1	Prmt7	-0.93	vasdef	G-EOD-22616
Esr1	Prmt8	-0.84	epididymus	G-EOD-22616
Esr1	Prmt8	-0.79	vasdef	G-EOD-22616
Esr1	Suv420h2	-0.83	epididymus	G-EOD-22616
Esr1	Suv420h2	-0.83	vasdef	G-EOD-22616
Esr2	Kdm2a	0.77	effduct	G-EOD-22616
Esr2	Kdm2a	0.90	testis	G-EOD-6881
Esr2	Setd1b	-0.77	testis	G-EOD-6881
Esr2	Setd1b	-0.83	effduct	G-EOD-22616

Nr0b2	Whsc1	0.78	testis	G-EOD-6881			
Nr0b2	Whsc1	0.91	testis	G-EOD-18211			
Nr1d1	Smyd3	0.82	epididymus	G-EOD-22616			
Nr1d1	Smyd3	0.77	vasdef	G-EOD-22616			
Nr1d2	Jmjd1c	-0.88	effduct	G-EOD-22616			
Nr1d2	Jmjd1c	-0.86	epididymus	G-EOD-22616			
Nr1d2	Prmt6	-0.85	testis	G-EOD-18211			
Nr1d2	Prmt6	-0.91	vasdef	G-EOD-22616			
Nr1h2	Carm1	0.88	epididymus	G-EOD-22616			
Nr1h2	Carm1	0.83	vasdef	G-EOD-22616			
Nr1h2	Ehmt1	0.76	epididymus	G-EOD-22616			
Nr1h2	Ehmt1	0.77	vasdef	G-EOD-22616			
Nr1h2	Ehmt2	0.98	testis	G-EOD-18211			
Nr1h2	Ehmt2	0.88	epididymus	G-EOD-22616			
Nr1h2	Ehmt2	0.88	vasdef	G-EOD-22616			
Nr1h2	Ezh2	0.85	vasdef	G-EOD-22616			
Nr1h2	Ezh2	0.95	epididymus	G-EOD-22616			
Nr1h2	Kdm4b	0.95	effduct	G-EOD-22616			
Nr1h2	Kdm4b	0.96	testis	G-EOD-18211			
Nr1h2	Setd8	0.90	testis	G-EOD-18211			
Nr1h2	Setd8	0.89	epididymus	G-EOD-22616			
Nr1h2	Suv420h2	0.96	epididymus	G-EOD-22616			
Nr1h2	Suv420h2	0.79	effduct	G-EOD-22616			
Nr1h2	Suv420h2	0.93	vasdef	G-EOD-22616			
Nr1h3	Smyd1	0.79	vasdef	G-EOD-22616			
Nr1h3	Smyd1	0.84	testis	G-EOD-18211			
Nr1h4	Mll2	0.84	testis	G-EOD-18211			
Nr1h4	Mll2	0.85	vasdef	G-EOD-22616			
Nr1h4	Setd1b	0.82	testis	G-EOD-18211			
Nr1h4	Setd1b	0.79	effduct	G-EOD-22616			
Nr1i2	Men1	0.86	testis	G-EOD-18211			
Nr1i2	Men1	0.83	vasdef	G-EOD-22616			
Nr1i3	Aof1	-0.91	effduct	G-EOD-22616			

Nr1i3	Aof1	-0.83	testis	G-EOD-18211		
Nr1i3	Eed	0.82	testis	G-EOD-18211		
Nr1i3	Eed	0.82	effduct	G-EOD-22616		
Nr1i3	Satb1	0.78	vasdef	G-EOD-22616		
Nr1i3	Satb1	0.75	effduct	G-EOD-22616		
Nr1i3	Whsc1l1	-0.88	testis	G-EOD-18211		
Nr1i3	Whsc1l1	-0.81	vasdef	G-EOD-22616		
Nr2c1	Ehmt2	0.83	epididymus	G-EOD-22616		
Nr2c1	Ehmt2	0.84	vasdef	G-EOD-22616		
Nr2c1	Kdm2b	0.78	testis	G-EOD-6881		
Nr2c1	Kdm2b	0.83	epididymus	G-EOD-22616		
Nr2c1	Mecp2	-0.87	testis	G-EOD-18211		
Nr2c1	Mecp2	-0.77	epididymus	G-EOD-22616		
Nr2c1	Smyd2	0.80	epididymus	G-EOD-22616		
Nr2c1	Smyd2	0.80	vasdef	G-EOD-22616		
Nr2c2	Ehmt1	-0.87	testis	G-EOD-18211		
Nr2c2	Ehmt1	-0.81	effduct	G-EOD-22616		
Nr2c2	Kdm5b	-0.84	testis	G-EOD-18211		
Nr2c2	Kdm5b	-0.77	effduct	G-EOD-22616		
Nr2c2	Nsd1	-0.78	effduct	G-EOD-22616		
Nr2c2	Nsd1	-0.77	vasdef	G-EOD-22616		
Nr2c2	Prdm5	-0.79	effduct	G-EOD-22616		
Nr2c2	Prdm5	-0.83	testis	G-EOD-18211		
Nr2e3	Baz2a	-0.80	effduct	G-EOD-22616		
Nr2e3	Baz2a	-0.90	testis	G-EOD-18211		
Nr2e3	Ctcf	0.75	epididymus	G-EOD-22616		
Nr2e3	Ctcf	0.90	effduct	G-EOD-22616		
Nr2e3	Ctcf	-0.94	vasdef	G-EOD-22616		
Nr2e3	Ctcf	-0.91	testis	G-EOD-18211		
Nr2e3	Kdm5a	0.85	epididymus	G-EOD-22616		
Nr2e3	Kdm5a	0.78	effduct	G-EOD-22616		
Nr2f6	Kdm4b	0.76	epididymus	G-EOD-22616		
Nr2f6	Kdm4b	0.83	testis	G-EOD-6881		

Nr3c1	Ehmt2	-0.84	testis	G-EOD-18211		
Nr3c1	Ehmt2	-0.79	epididymus	G-EOD-22616		
Nr3c1	Kdm2a	-0.81	effduct	G-EOD-22616		
Nr3c1	Kdm2a	-0.96	testis	G-EOD-18211		
Nr3c1	Smyd2	-0.82	testis	G-EOD-18211		
Nr3c1	Smyd2	-0.75	epididymus	G-EOD-22616		
Nr3c1	Suv39h1	-0.82	testis	G-EOD-18211		
Nr3c1	Suv39h1	-0.83	vasdef	G-EOD-22616		
Nr3c2	Dnmt3b	0.87	testis	G-EOD-18211		
Nr3c2	Dnmt3b	0.83	vasdef	G-EOD-22616		
Nr3c2	Ehmt2	-0.81	effduct	G-EOD-22616		
Nr3c2	Ehmt2	-0.80	epididymus	G-EOD-22616		
Nr3c2	Kdm3a	0.92	testis	G-EOD-18211		
Nr3c2	Kdm3a	0.79	vasdef	G-EOD-22616		
Nr4a1	Paxip1	0.81	vasdef	G-EOD-22616		
Nr4a1	Paxip1	0.89	testis	G-EOD-6881		
Nr4a1	Prmt8	-0.90	testis	G-EOD-18211		
Nr4a1	Prmt8	-0.80	epididymus	G-EOD-22616		
Nr4a1	Whsc1	0.78	epididymus	G-EOD-22616		
Nr4a1	Whsc1	0.84	testis	G-EOD-6881		
Nr4a2	Dot1l	-0.83	vasdef	G-EOD-22616		
Nr4a2	Dot1l	-0.83	epididymus	G-EOD-22616		
Nr4a2	Ehmt1	-0.83	vasdef	G-EOD-22616		
Nr4a2	Ehmt1	-0.82	epididymus	G-EOD-22616		
Nr4a2	Ehmt2	-0.90	testis	G-EOD-18211		
Nr4a2	Ehmt2	-0.90	epididymus	G-EOD-22616		
Nr4a2	Kdm2b	-0.80	vasdef	G-EOD-22616		
Nr4a2	Kdm2b	-0.88	epididymus	G-EOD-22616		
Nr4a2	Kdm4b	-0.87	epididymus	G-EOD-22616		
Nr4a2	Kdm4b	-0.97	testis	G-EOD-18211		
Nr4a2	Satb1	-0.95	vasdef	G-EOD-22616		
Nr4a2	Satb1	-0.92	epididymus	G-EOD-22616		
Nr4a2	Setd8	0.84	testis	G-EOD-6881		

Nr4a2	Setd8	0.91	effduct	G-EOD-22616			
Nr4a2	Smyd1	-0.78	vasdef	G-EOD-22616			
Nr4a2	Smyd1	-0.84	testis	G-EOD-18211			
Nr4a2	Smyd2	-0.86	testis	G-EOD-18211			
Nr4a2	Smyd2	-0.83	epididymus	G-EOD-22616			
Nr4a3	Dpy30	-0.90	testis	G-EOD-18211			
Nr4a3	Dpy30	-0.89	vasdef	G-EOD-22616			
Nr4a3	Smyd3	-0.78	vasdef	G-EOD-22616			
Nr4a3	Smyd3	-0.84	epididymus	G-EOD-22616			
Nr5a1	Kdm5c	0.84	effduct	G-EOD-22616			
Nr5a1	Kdm5c	0.99	testis	G-EOD-18211			
Nr5a1	Paxip1	-0.82	vasdef	G-EOD-22616			
Nr5a1	Paxip1	-0.77	testis	G-EOD-6881			
Nr6a1	Ezh2	0.85	vasdef	G-EOD-22616			
Nr6a1	Ezh2	0.84	epididymus	G-EOD-22616			
Nr6a1	Kdm5c	-0.92	testis	G-EOD-18211			
Nr6a1	Kdm5c	-0.77	vasdef	G-EOD-22616			
Pgr	Cxxc1	-0.95	testis	G-EOD-18211			
Pgr	Cxxc1	-0.88	effduct	G-EOD-22616			
Pgr	Kdm6b	0.93	epididymus	G-EOD-22616			
Pgr	Kdm6b	0.91	testis	G-EOD-18211			
Pgr	Mll3	0.76	effduct	G-EOD-22616			
Pgr	Mll3	0.86	testis	G-EOD-18211			
Ppara	Bcor	-0.80	effduct	G-EOD-22616			
Ppara	Bcor	-0.77	epididymus	G-EOD-22616			
Ppard	Ash2l	0.87	vasdef	G-EOD-22616			
Ppard	Ash2l	0.83	testis	G-EOD-6881			
Ppard	Bcor	-0.82	testis	G-EOD-18211			
Ppard	Bcor	-0.77	vasdef	G-EOD-22616			
Ppard	Bcor	-0.79	epididymus	G-EOD-22616			
Ppard	Ehmt1	0.87	epididymus	G-EOD-22616			
Ppard	Ehmt1	0.86	vasdef	G-EOD-22616			
Ppard	Ehmt2	0.85	epididymus	G-EOD-22616			

Ppard	Ehmt2	0.96	vasdef	G-EOD-22616			
Ppard	Ezh2	0.89	epididymus	G-EOD-22616			
Ppard	Ezh2	0.90	vasdef	G-EOD-22616			
Ppard	Kdm4b	0.88	testis	G-EOD-6881			
Ppard	Kdm4b	0.80	epididymus	G-EOD-22616			
Ppard	Kdm4c	-0.75	epididymus	G-EOD-22616			
Ppard	Kdm4c	-0.95	testis	G-EOD-18211			
Ppard	Kdm6a	-0.85	testis	G-EOD-18211			
Ppard	Kdm6a	-0.91	vasdef	G-EOD-22616			
Ppard	Mecp2	-0.82	epididymus	G-EOD-22616			
Ppard	Mecp2	-0.81	vasdef	G-EOD-22616			
Ppard	Prmt7	0.78	vasdef	G-EOD-22616			
Ppard	Prmt7	0.81	epididymus	G-EOD-22616			
Ppard	Prmt8	0.88	vasdef	G-EOD-22616			
Ppard	Prmt8	0.85	epididymus	G-EOD-22616			
Ppard	Suv420h2	0.89	epididymus	G-EOD-22616			
Ppard	Suv420h2	0.90	vasdef	G-EOD-22616			
Pparg	Setd8	0.92	testis	G-EOD-18211			
Pparg	Setd8	0.88	epididymus	G-EOD-22616			
Rara	Cxxc1	0.88	vasdef	G-EOD-22616			
Rara	Cxxc1	0.80	epididymus	G-EOD-22616			
Rara	Eed	-0.87	epididymus	G-EOD-22616			
Rara	Eed	-0.83	effduct	G-EOD-22616			
Rara	Kdm4a	-0.82	effduct	G-EOD-22616			
Rara	Kdm4a	-0.86	testis	G-EOD-18211			
Rara	Prdm5	-0.90	epididymus	G-EOD-22616			
Rara	Prdm5	-0.91	vasdef	G-EOD-22616			
Rarb	Ash2l	0.85	epididymus	G-EOD-22616			
Rarb	Ash2l	0.82	testis	G-EOD-18211			
Rarb	Kdm5d	0.99	testis	G-EOD-18211			
Rarb	Kdm5d	0.88	effduct	G-EOD-22616			
Rarb	Suv39h1	0.87	effduct	G-EOD-22616			
Rarb	Suv39h1	0.93	vasdef	G-EOD-22616			

Rarb	Suv39h1	0.78	epididymus	G-EOD-22616			
Rarg	Aof1	0.75	epididymus	G-EOD-22616			
Rarg	Aof1	0.91	vasdef	G-EOD-22616			
Rarg	Bcor	-0.82	vasdef	G-EOD-22616			
Rarg	Bcor	-0.78	epididymus	G-EOD-22616			
Rarg	Cxxc1	0.93	testis	G-EOD-18211			
Rarg	Cxxc1	0.77	epididymus	G-EOD-22616			
Rarg	Ehmt2	0.95	vasdef	G-EOD-22616			
Rarg	Ehmt2	0.85	epididymus	G-EOD-22616			
Rarg	Prdm2	0.87	testis	G-EOD-18211			
Rarg	Prdm2	0.80	epididymus	G-EOD-22616			
Rarg	Prmt7	0.91	vasdef	G-EOD-22616			
Rarg	Prmt7	0.96	epididymus	G-EOD-22616			
Rora	Kdm1	-0.76	vasdef	G-EOD-22616			
Rora	Kdm1	-0.88	testis	G-EOD-18211			
Rorb	Dot1l	-0.93	effduct	G-EOD-22616			
Rorb	Dot1l	-0.82	testis	G-EOD-18211			
Rorb	Kdm6b	-0.91	testis	G-EOD-18211			
Rorb	Kdm6b	-0.92	effduct	G-EOD-22616			
Rxra	Baz2a	0.76	effduct	G-EOD-22616			
Rxra	Baz2a	0.77	testis	G-EOD-6881			
Rxra	Ehmt2	0.75	effduct	G-EOD-22616			
Rxra	Ehmt2	0.78	epididymus	G-EOD-22616			
Rxra	Kdm2b	0.89	epididymus	G-EOD-22616			
Rxra	Kdm2b	0.87	testis	G-EOD-6881			
Rxra	Kdm5d	-0.85	testis	G-EOD-6881			
Rxra	Kdm5d	-0.87	vasdef	G-EOD-22616			
Rxra	Kdm5d	-0.86	testis	G-EOD-18211			
Rxra	Satb1	0.78	epididymus	G-EOD-22616			
Rxra	Satb1	0.76	vasdef	G-EOD-22616			
Rxrb	Carm1	0.83	epididymus	G-EOD-22616			
Rxrb	Carm1	0.75	vasdef	G-EOD-22616			
Rxrb	Jmjd6	-0.84	epididymus	G-EOD-22616			

Rxrb	Jmjd6	-0.84	vasdef	G-EOD-22616		
Rxrb	Mll2	0.87	testis	G-EOD-18211		
Rxrb	Mll2	0.77	vasdef	G-EOD-22616		
Rxrb	Paxip1	0.79	vasdef	G-EOD-22616		
Rxrb	Paxip1	0.87	effduct	G-EOD-22616		
Rxrb	Suv420h2	0.85	testis	G-EOD-18211		
Rxrb	Suv420h2	0.86	epididymus	G-EOD-22616		
Rxrg	Kdm1	0.91	testis	G-EOD-18211		
Rxrg	Kdm1	0.79	epididymus	G-EOD-22616		
Rxrg	Kdm2b	0.85	effduct	G-EOD-22616		
Rxrg	Kdm2b	0.89	epididymus	G-EOD-22616		
Rxrg	Kdm2b	0.83	vasdef	G-EOD-22616		
Rxrg	Mll1	-0.80	vasdef	G-EOD-22616		
Rxrg	Mll1	-0.89	epididymus	G-EOD-22616		
Rxrg	Nsd1	-0.93	epididymus	G-EOD-22616		
Rxrg	Nsd1	-0.95	vasdef	G-EOD-22616		
Rxrg	Smyd2	0.87	testis	G-EOD-18211		
Rxrg	Smyd2	0.91	epididymus	G-EOD-22616		
Thra	Carm1	0.86	vasdef	G-EOD-22616		
Thra	Carm1	0.81	testis	G-EOD-18211		
Thra	Dnmt3b	0.88	vasdef	G-EOD-22616		
Thra	Dnmt3b	0.79	epididymus	G-EOD-22616		
Thra	Kdm4c	-0.89	testis	G-EOD-18211		
Thra	Kdm4c	-0.83	epididymus	G-EOD-22616		
Thra	Mll1	0.84	testis	G-EOD-6881		
Thra	Mll1	0.82	effduct	G-EOD-22616		
