# Supplementary material for: Coexpression of Nuclear Receptors and Histone Methylation Modifying Genes in the Testis: Implications for Endocrine Disruptor Modes of Action
Source: PLoS One. 2012 Apr 4;7(4):e34158. doi: 10.1371/journal.pone.0034158 (PMC3319570; doi:10.1371/journal.pone.0034158)
Supplement: Table S4 — Data sources. (PDF) [file pone.0034158.s005.pdf]

Supplementary Table 4: Data sources

Experiment 1: Accession No - E-TABM-130 Platform - Human Genome U133 Plus 2.0 (Chalmel F, et al, 2007, Proc Natl Acad Sci U S A 104: 8346-8351.)

This dataset was selected to allow comparison of coexpression profiles across species. Samples include: 8 human seminiferous tubules, total testis, spermatocytes and spermatid cells pooled from several adult individuals; 12 mouse seminiferous tubules, total testis, spermatocytes and spermatid cells pooled from several adult individuals; 12 rat seminiferous tubules, total testis, spermatocytes and spermatid cells pooled from several juvenile or adult Sprague-Dawley rats.

Experiment 2: Accession No - E-GEOD-10919 Platform - Rat Genome 230 2.0 (Anway MD, et al, 2008, Genomics 91: 30-40.)

This trans-generational study provided testis samples collected at embryonic day 16 from Sprague-Dawley rats. 2 controls and 2 from F0 vinclozolin exposed dam (100mg/kg body weight) from F1 through to F3 generations. This dataset was selected due to the relevance of the chemical and the exposure time point to endocrine disruption.

Experiment 3: Accession No - E-GEOD-25196 Platform - Rat Genome 230 2.0 (Johnson K, et al 2011, Toxicol Sci 120: 460-474.)

Fischer 344 rats were exposed via oral gavage of the dam to vehicle (corn oil, n= 4) or 50 mg/kg (body weight) dibutyl phthalate (DBP n= 4) daily from gestational day (GD) 12 to 20. Samples were collected six hours after the final exposure on GD20. Again this dataset was selected for relevance of the chemical and exposure point to endocrine disruption.

Experiment 4: Accession No - E-GEOD-13550 Platform - Rat Genome 230 2.0

This dataset was submitted to ArrayExpress by the same author as Experiment 3. Fischer 344 rats were exposed in utero to 50mg/kg body weight dibutyl phthalate (n=4) or control vehicle (n =4) from gestational day 12 to 20, as per experiment 3, however samples were not collected until post natal day 35.

Experiment 5: Accession No - E-GEOD-20952 Platform - Rat Genome 230 2.0 (Yuan X, 2011 J Appl Toxicol 31: 421-430.)

10 weeks old male rats were treated with a single dose of either reprotoxic or non-reprotoxic phthalates and sacrificed 24h thereafter. 5 controls and 5 samples exposed to dibutyl phthalate (200mg/kg body weight) were selected for inclusion in the current study to allow profiling of the genes of interest in adult tissue following exposure to a phthalate compound.

Experiment 6: Accession No - E-GEOD-10412 Platform - Rat Genome 230 2.0

This experiment was conducted by D. Dix at the U.S. Environmental Protection Agency. The dataset contains testis samples from Wister Han IGS rats fed low or high doses of myclobutanil, propiconazole, triadimefon or vehicle control from gestation day six to postnatal day 92 and control samples. This dataset was selected to allow comparison of expression patterns for genes of interest following exposure to different types of compounds in the testis.

Experiment 7: Accession No - E-GEOD-22616 Platform - Mouse Genome 430 2.0 (Snyder EM, 2010, Dev Dynam 239:2479-2491)

This dataset contains samples from the efferent ducts, epididymis and vas deferens from tissues collected at four time points: E14.5, E16.5, E18.5 and postnatal day 1. Total samples = 24, 8 per tissue, 2 per time point. This dataset was selected to allow profiling of the genes of interest in different tissues of the testis.

Experiment 8: Accession No - E-GEOD-18211 Platform - Mouse Genome 430 2.0

This dataset comprised somatic support cells collected at embryonic day 11.5 and 12.5 during gonadal development and differentiation of ovary and testis that were harveted to provide spatial-temporal expression patterns of genes underlying gonadal development. This dataset contains 6 samples from ovary and six from testis. The testis samples were included in the current analysis to increase the number of embryonic samples for analysis

Experiment 9: Accession No - E-GEOD-6881 Platform - Mouse Genome 430 2.0 (Small C, 2005, Biol Reprod 72: 492-501.)

This time course experiment was conducted to profile gene expression during the development of the murine embryonic gonad. Samples n= 10, two replicates for each of five time points: E11.5, E12.5, E14.5, E16.5, E18.5. This dataset was selected to allow comparison between coexpression patterns in rat and mouse embryonic tissue and between mouse embryonic and adult tissue.

Experiment 10: Accession No - E-GEOD- 29963 Platform - Rat Genome 230 2.0 (Paul C, 2011, Biol Reprod 85: 1269-1278.)

Spermatocytes and round spermatids were isolated from the testes of young (4 months) and aged (18 months) rats. The dataset contains a total of 24 samples with six replicates for each cell type and age group. These data were selected for the current study to provide insight into the coexpression of the genes of interest within specific cell types as opposed to whole testis tissue.
